# Mycobacterium tuberculosis Transcription Factor EmbR Regulates the Expression of Key Virulence Factors That Aid in *Ex Vivo* and *In Vivo* Survival

**DOI:** 10.1128/mbio.03836-21

**Published:** 2022-04-26

**Authors:** Suresh Kumar, Mehak Zahoor Khan, Neha Khandelwal, Chen Chongtham, Biplab Singha, Ankita Dabla, Debashree Behera, Archana Singh, Balasubramanian Gopal, G. Aneeshkumar Arimbasseri, Siddhesh S. Kamat, Vinay Kumar Nandicoori

**Affiliations:** a National Institute of Immunology, New Delhi, India; b Department of Biology, Indian Institute of Science Education and Research, Pune, India; c Academy of Scientific and Innovative Research (AcSIR), Ghaziabad, India; d Molecular Biophysics Unit, Indian Institute of Science, Bangalore, India; Washington University School of Medicine in St. Louis

**Keywords:** transcription, transcription factors, hypoxia, mycobacteria, tuberculosis, granuloma, EmbR, *Mycobacterium tuberculosis*

## Abstract

Mycobacterium tuberculosis encodes ~200 transcription factors that modulate gene expression under different microenvironments in the host. Even though high-throughput chromatin immunoprecipitation sequencing and transcriptome sequencing (RNA-seq) studies have identified the regulatory network for ~80% of transcription factors, many transcription factors remain uncharacterized. EmbR is one such transcription factor whose *in vivo* regulon and biological function are yet to be elucidated. Previous *in vitro* studies suggested that phosphorylation of EmbR by PknH upregulates the *embCAB* operon. Using a gene replacement mutant of *embR*, we investigated its role in modulating cellular morphology, antibiotic resistance, and survival in the host. Contrary to the prevailing hypothesis, under normal growth conditions, EmbR is neither phosphorylated nor impacted by ethambutol resistance through the regulation of the *embCAB* operon. The *embR* deletion mutant displayed attenuated M. tuberculosis survival *in vivo*. RNA-seq analysis suggested that EmbR regulates operons involved in the secretion pathway, lipid metabolism, virulence, and hypoxia, including well-known hypoxia-inducible genes *devS* and *hspX*. Lipidome analysis revealed that EmbR modulates levels of all lysophospholipids, several phospholipids, and M. tuberculosis-specific lipids, which is more pronounced under hypoxic conditions. We found that the EmbR mutant is hypersusceptible to hypoxic stress, and RNA sequencing performed under hypoxic conditions indicated that EmbR majorly regulates genes involved in response to acidic pH, hypoxia, and fatty acid metabolism. We observed condition-specific phosphorylation of EmbR, which contributes to EmbR-mediated transcription of several essential genes, ensuring enhanced survival. Collectively, the study establishes EmbR as a key modulator of hypoxic response that facilitates mycobacterial survival in the host.

## INTRODUCTION

Despite its existence since antiquity, tuberculosis still ranks among the foremost killers of the 21st century. Upon infection, Mycobacterium tuberculosis is phagocytosed by the alveolar macrophages, often remodeling the site of infection into granuloma (1). M. tuberculosis can survive in the latent state inside the granuloma in 90% of the cases or form active disease in ~10% of the infected individuals. The ability to survive under various host-induced stresses, such as reactive nitrogen intermediates (RNI), reactive oxygen species (ROS), nutrient starvation, and hypoxia, requires more profound insight into mechanisms adopted by M. tuberculosis. In response to these stresses, the bacilli induce transcriptional modulation, resulting in the secretion of virulence factors, metabolic changes, induction of stress response genes, etc. This helps in adaptation and persistence inside the host, resulting in the infection’s establishment (2–7). Thus, it is imperative to investigate the effectors of these signaling pathways that aid in bacillary survival inside the host. These effectors’ expression is often regulated by a plethora of transcription factors (TFs) (8–10).

M. tuberculosis genome encodes ~214 TFs, which regulate gene expression under various conditions and stages of its life cycle. Studies have found ~16,000 binding events from 154 TFs from the M. tuberculosis genome, underlining the importance of transcriptional regulation in M. tuberculosis. A total of 5,400 of 15,980 TF binding sites (approximately one-third) are present within the 220-bp promoter window, which has resulted in 47,200 TF–promoter interactions (11, 12). TFs undergo a range of posttranslational modifications (PTMs) that orchestrate their transcriptional activity (13). Phosphorylation is a more common and prominent PTM than sumoylation, methylation, acetylation, glycosylation, etc. M. tuberculosis encodes 11 serine threonine protein kinases (STPKs) that phosphorylate various TFs (14, 15).

**FIG 1:**
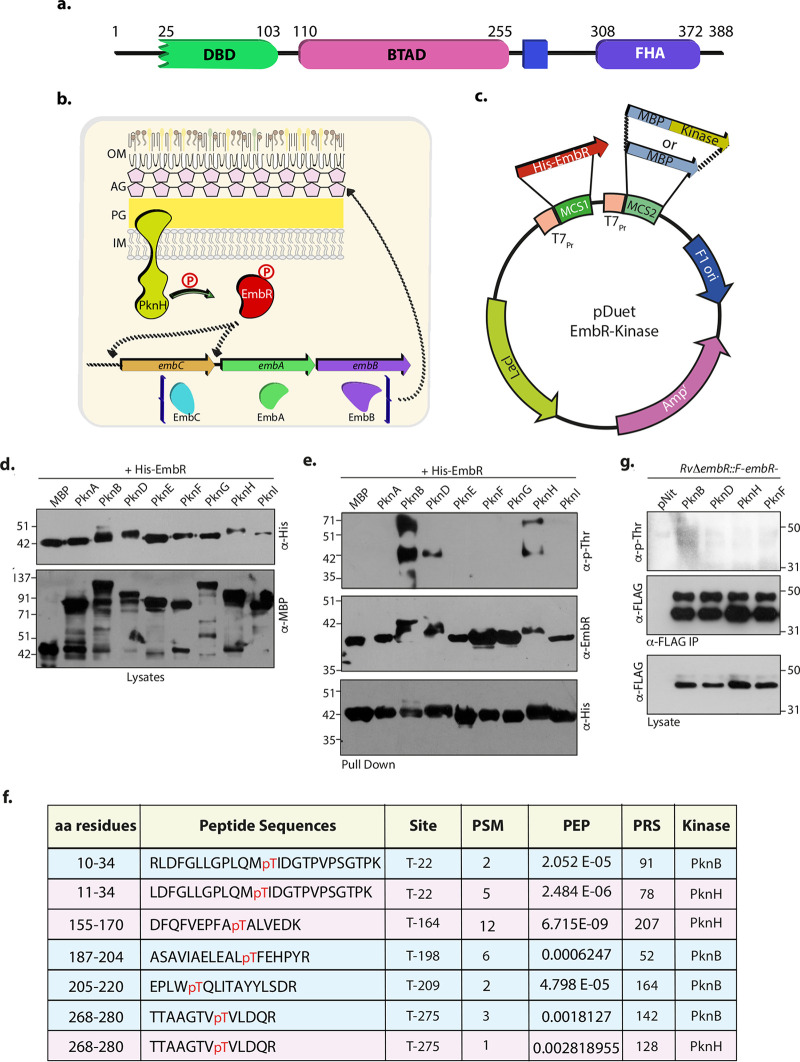
EmbR is phosphorylated *in vitro* but not *in vivo* under regular growth conditions. (a) Schematic depicting domain architecture of EmbR in M. tuberculosis. (b) Model demonstrating previously known roles of EmbR. Phosphorylation of EmbR by PknH results in enhanced transcription of *embCAB* operon, modulating AG layer biosynthesis. OM, outer membrane; AG, arabinogalactan layer; PG, peptidoglycan layer; IM, inner membrane. (c) Schematic representation of the pDuetEmbR-kinase vectors used for the dual expression of *embR* and M. tuberculosis STPKs. Vectors with different STPKs in MCS II with MBP (Maltose Binding Protein) tag and *embR* with N-terminal hexa-His tag were transformed in the surrogate host *E. coli* BL21 cells, and the expression of EmbR- and MBP-tagged kinases was induced with 0.1 mM isopropyl-β-d-thiogalactopyranoside (IPTG). (d) Western blot analysis of lysates confirming coexpression of EmbR and STPKs with anti-His (upper) and anti-MBP (lower), respectively. (e) His-EmbR was immunoprecipitated (IP) using Ni^2+^ affinity agarose beads from various pDuet-EmbR-Kinase strains. The pulldowns were probed with α-p-Thr (upper) to assess EmbR phosphorylation and with α-EmbR and α-His antibodies as controls. (f) Table summarizing five phosphopeptides obtained from PknB- and PknH-targeted *E. coli* EmbR using MS/MS. T-164 and T-198/T-209 are unique to PknH and PknB, respectively. T-22 and T-275 are common target sites from both PknB and PknH. PSM, peptide-spectrum match; PEP, posterior error probability; PRS, pattern recognition for spectra; aa, amino acid. (g) M. tuberculosis *RvΔembR*::*F-embR* strain was electroporated with pNit-PknB, pNit-PknD, pNit-PknE, and pNit-PknF. The resulting strains were induced for the kinase overexpression with 5 μM isovaleronitrile (IVN), and the WCLs were probed with α-FLAG antibody. The lysates were immunoprecipitated (IP) with FLAG-M2 beads; 1/10 of the IP was probed with α-FLAG, and the remaining 9/10 of the IP was probed with α-p-Thr antibodies.

*embR* (Rv1267c) is one such transcription factor, belonging to the family of *Streptomyces* antibiotic regulatory proteins (SARP) that is located in the same operon, upstream of the *pknH* gene (Rv1266c) (16). The crystal structure of EmbR showed three distinct domains: N-terminal OmpR/PhoB-like DNA binding domain (DBD), consisting of winged-like structure for the binding; middle central all-helical bacterial transcriptional activation domain (BTAD), hypothesized to aid in the DNA binding; and the C-terminal fork head-associated (FHA) domain (Fig. 1a) (17). FHA domain is essential for its interaction and phosphorylation by PknH and results in enhanced transcription of *embCAB* genes (18–20). In addition to PknH, EmbR is phosphorylated *in vitro* by PknA, PknB, PknE, and PknF (21, 22). The augmented transcription of *embC* increases the lipoarabinomannan/lipomannan (LAM/LM) ratio, modulating the arabinogalactan (AG) layer (Fig. 1b) (19). Furthermore, mutations in *embCAB* and *embR* have been linked to ethambutol (EMB) resistance in various clinical isolates, highlighting the importance of EmbR-mediated regulation in M. tuberculosis virulence and pathogenesis (23, 24).

In this study, we aimed to validate previously known indirect *in vitro* evidence with the help of a gene replacement mutant and asked the following questions. (i) What is the biological function of EmbR? (ii) What are the genes that EmbR regulates? (iii) Does EmbR promote the survival of M. tuberculosis inside the host? Our results show that EmbR deletion mutant displayed attenuated M. tuberculosis survival inside the murine peritoneal macrophages and *in vivo*. Scanning electron microscopy (SEM), transmission electron microscopy (TEM), and expression analysis suggested that EmbR modulates cellular length and cell wall ultrastructure but not through *embCAB*. Among all the *in vitro* stress experiments, mutant demonstrated reduced survival, specifically in modified Wayne’s hypoxia model. RNA sequencing data showed downregulation of operons involved in the secretion pathways, lipid metabolism, virulence, and hypoxia establishment in the mutant. Lipidome analysis suggested that EmbR plays a crucial role in cell wall lipid composition during normoxia and hypoxia. Furthermore, RNA sequencing of hypoxic cultures indicated condition-specific regulation of several essential genes, including those involved in fatty acid metabolism, response to acidic pH, and hypoxia. Interestingly, while we could not detect phosphorylation of EmbR under normal growth conditions, it is robustly phosphorylated under acidic and hypoxic conditions. Together, this study identifies novel downstream targets of EmbR and establishes EmbR as a crucial regulatory protein in determining M. tuberculosis virulence and pathogenesis.

## RESULTS

### EmbR is phosphorylated *in vitro* but not *in vivo* under regular growth conditions.

Based on the *in vitro* kinase assays, EmbR has been demonstrated to be a substrate for serine/threonine protein kinases PknA, PknB, PknE, PknF, and PknH (20–22). However, the target phosphorylation sites for these kinases have not been identified. We sought to utilize Escherichia coli as the surrogate host and a previously developed pDUET vector system to identify the target sites. Either MBP (Maltose Binding Protein) or MBP-tagged full-length or kinase domains of all 11 M. tuberculosis STPKs were cloned into the second MCS (multiple cloning site). EmbR was cloned into the unique HindIII site in the first MCS, and the constructs were transformed into E. coli BL21 Codon Plus strain (Fig. 1c). We observed robust expression of EmbR and MBP or MBP-tagged kinases for 8 out of 11 kinases (Fig. 1d). To evaluate the phosphorylation status of EmbR, His-tagged EmbR was pulled down with the help of Ni^2+^ affinity beads and probed with α-p-Thr and α-EmbR antibodies. In α-EmbR blots, we observed higher-molecular-mass band(s) when EmbR was coexpressed with PknB, PknD, and PknH. Consonantly, α-p-Thr blots indicated that this higher-molecular-mass band(s) corresponds to pEmbR (Fig. 1e), suggesting that PknB, PknD, and PknH robustly phosphorylate EmbR. The phosphorylation sites on EmbR for kinases PknB and PknH were identified by excising EmbR band, followed by trypsinization and subjecting the tryptic peptides to liquid chromatography coupled to mass spectrometric (LC-MS) analysis. We identified five target sites on EmbR for PknB and PknH, of which T164 is a unique target site for PknH, while PknB exclusively phosphorylated T198 and T209. T22 and T275 were phosphorylated by both PknB and PknH (Fig. 1f; see also Fig. S1 and S2 in the supplemental material). Subsequently, we sought to delineate the role of PknB- and PknH-mediated phosphorylation on the function of EmbR. Toward this end, we first sought to establish if EmbR is indeed phosphorylated *in vivo*. M. tuberculosis *RvΔembR* mutant was coelectroporated with pF-F-embR and pNit-PknB/PknD/PknH constructs. Expression of kinases was induced, and FLAG-tagged EmbR was immunoprecipitated, resolved, and probed with α-FLAG and α-p-Thr antibodies. To our surprise, we did not detect any specific band corresponding to pEmbR (Fig. 1g). FLAG-EmbR was excised from the gel and trypsinized, and the peptides were subjected to LC-MS analysis to confirm the results further. LC-MS experiment did not detect any phosphorylated peptides, suggesting that EmbR is not phosphorylated in M. tuberculosis under regular growth conditions.

10.1128/mbio.03836-21.6 FIG S1 MS/MS spectra of PknB target EmbR phosphopeptides. Download FIG S1, TIF file, 1.2 MB. Copyright © 2022 Kumar et al. 2022 Kumar et al. https://creativecommons.org/licenses/by/4.0/ This content is distributed under the terms of the Creative Commons Attribution 4.0 International license.

### Deletion of EmbR does not impact M. tuberculosis growth *in vitro*.

**FIG 2:**
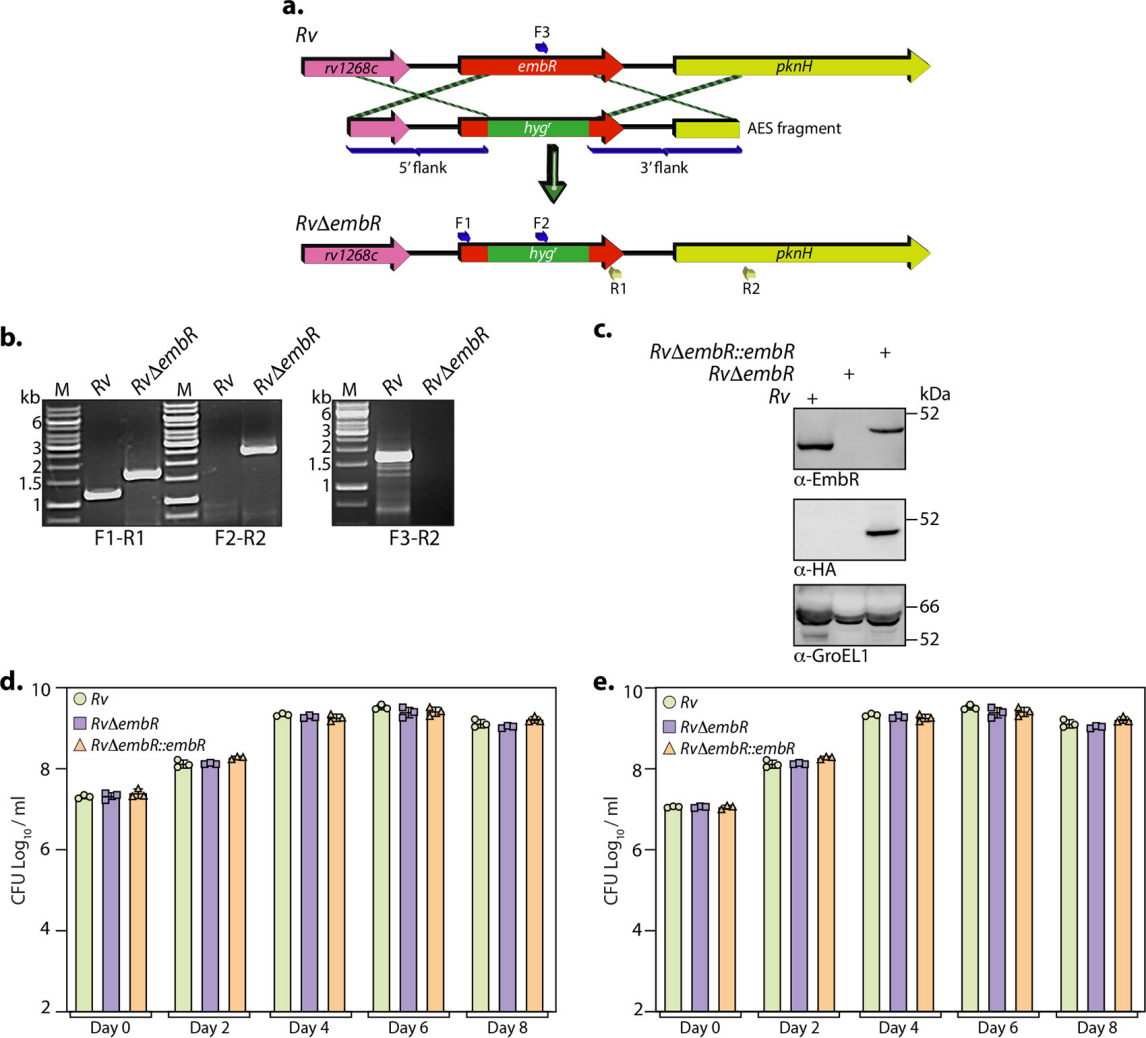
Generation and characterization of *RvΔembR* mutant. (a) Pictorial representation of the genetic organization of *embR* loci in *Rv* and *RvΔembR* mutant. The figure depicts the replacement of *embR* with *hyg^r^*. Primer sets used for validating the generation of mutant strain are indicated. (b) The deletion of *embR* at its native locus was confirmed with the help of PCRs using different sets of primers. The first panel shows PCR amplicons with a gene-specific primer set (F1-R1) in *Rv* (1,147 bp) and *RvΔembR* mutant (1,728 bp). The second panel shows amplicons (F2-R2) expected only in *RvΔembR* mutant (2,549 bp), and the third panel shows amplicons (F3-R2) expected only in *Rv* (1703 bp). M represents 1-kb gene ruler ladder. (c) A total of 30 μg of WCLs prepared from *Rv*, *RvΔembR*, and *RvΔembR*::*embR* strains was resolved, transferred to the nitrocellulose membrane, and probed with α-EmbR (upper), α-HA (middle), and α-GroEL1 (lower) antibodies. (d and e) *Rv*, *RvΔembR*, and *RvΔembR*::*embR* strains were inoculated at an OD_600_ of ~0.1 in 7H9-ADC (d) and Sauton’s (e) medium. The bacillary survival was monitored by CFU enumeration at indicated time points. Data are presented as mean CFU log_10_/mL ± standard deviations (SD) and are representative of two biologically independent experiments, each performed in triplicates (*n* = 3).

EmbR comes from an *in vitro* nonessential gene in Himar1 transposon mutagenesis (25, 26). To investigate the role of EmbR to modulate *embCAB* operon expression and its impact on the pathogen’s survival *in vivo*, we sought to generate an *embR* gene replacement mutant. Linearized allelic exchange substrate (AES) was electroporated into recombineering-proficient M. tuberculosis H37Rv strain (Rv; note that genotype is used as the strain name throughout the manuscript). Recombinants obtained were confirmed for the replacement of *embR* with *hyg^r^* selectable marker at its native locus by PCRs with three sets of primers using the genomic DNA isolated from wild-type Rv and *RvΔembR* mutant (Fig. 2a and b). pF-embR-HA construct was electroporated into *RvΔembR* mutant to generate *RvΔembR*::*embR* complementation strain. Western blot analysis of lysates prepared from *Rv* and *RvΔembR* strains further confirmed the deletion of *embR*. Notably, the expression of EmbR in the *RvΔembR*::*embR* mutant was similar to that of endogenous expression (Fig. 2c). Next, we evaluated the impact of *embR* deletion on the *in vitro* survival of M. tuberculosis. The cultures from *Rv*, *RvΔembR*, and *RvΔembR*::*embR* strains were grown in nutrient-rich 7H9 medium and limited Sauton’s medium. No significant differences were observed in *RvΔembR* strain compared with *Rv* and *RvΔembR*::*embR* strains (Fig. 2d and e), which led us to conclude that the deletion of EmbR does not affect the *in vitro* growth of mycobacteria.

### EmbR-mediated changes in mycobacterial cell wall architecture are not through *embCAB*.

**FIG 3:**
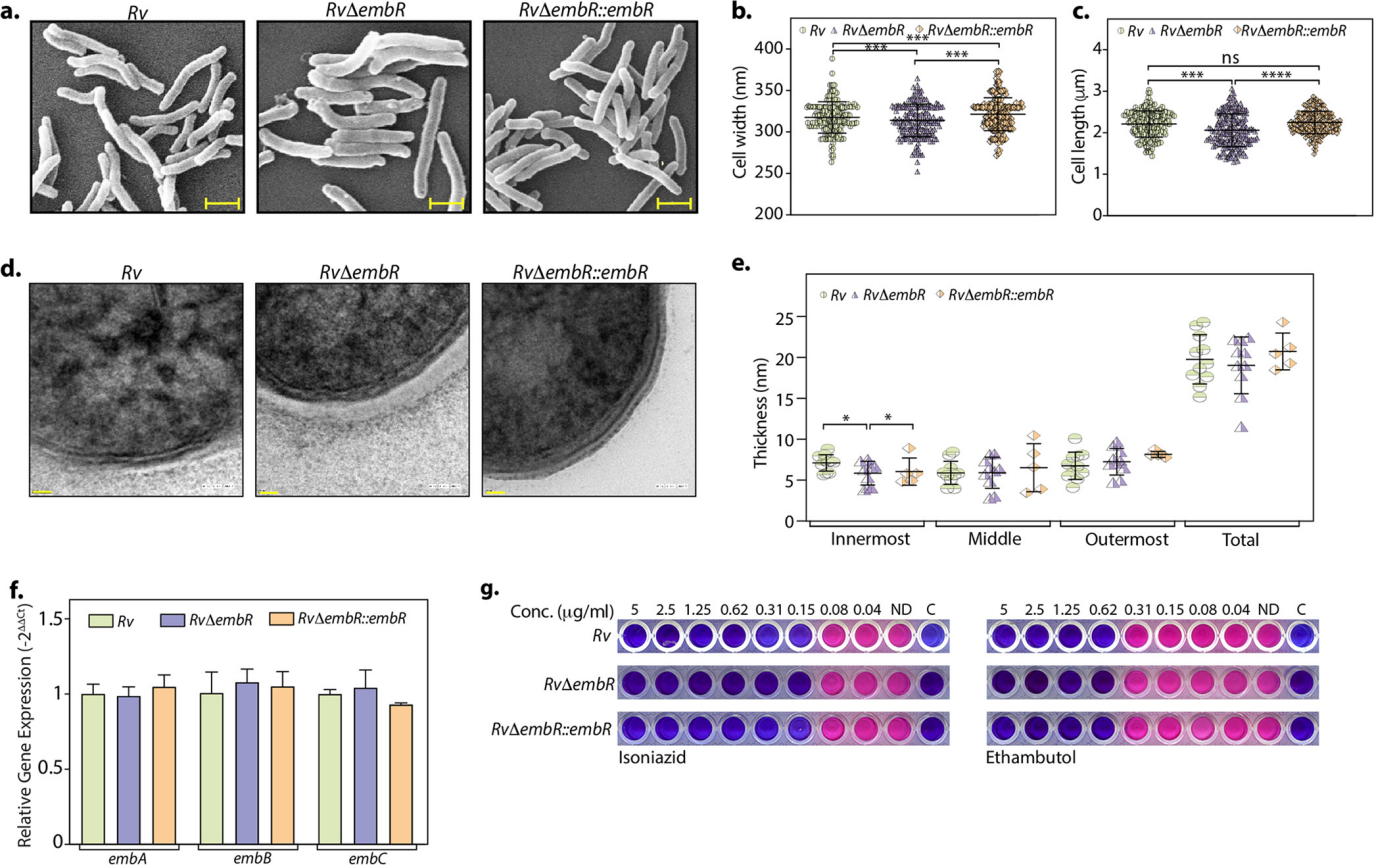
EmbR does not influence mycobacterial cell wall architecture. (a to c) Cultures of *Rv*, *RvΔembR*, and *RvΔembR*::*embR* strains were grown until an OD_600_ of ~0.6 in 7H9-ADC medium and fixed. (a) Cell morphology was observed through SEM at 15,000×. Scale bar, 1.0 μm. Cell width (b) and length (c) were quantified by the Smart Tiff program and plotted. (d) Fresh cultures of *Rv*, *RvΔembR*, and *RvΔembR*::*embR* strains were grown until an OD_600_ of ~0.6, fixed, and processed for TEM. Cell wall architecture was observed at 200 kV, 50,000×. (e) Quantification of innermost, middle, outermost, and total layer thickness. (f) Gene expression of *embCAB* operon was quantified in *Rv* and *RvΔembR* strains using qRT-PCR. (g) MICs of isoniazid and ethambutol were determined in *Rv*, *RvΔembR*, and *RvΔembR*::*embR* strains as described in Materials and Methods.

Electrophoretic mobility shift assays with the promoter regions of *embC*, *embA*, and *embB* and purified EmbR and pEmbR suggested that (i) it binds to the promoter region and (ii) the binding is improved upon its phosphorylation (21). Since EmbA and EmbB proteins are involved in the biosynthesis of arabinogalactan (AG), a significant cell wall component, we evaluated the impact of EmbR deletion on the cellular architecture. Toward this end, we performed scanning electron microscopy (SEM) and observed significant changes in cell width and length upon deletion of EmbR (Fig. 3a to c). We then evaluated the cell wall ultrastructure of *Rv*, *RvΔembR*, and *RvΔembR*::*embR* strains using transmission electron microscopy (TEM). The results demonstrated that while the mycolic acid layer (outermost) and the arabinogalactan (middle) were comparable in all three strains, peptidoglycan (innermost) was marginally thinner upon deletion of EmbR (Fig. 3d and e). Next, we investigated whether cell length and width changes are due to putative regulation of *embCAB.* To validate this, we examined the role of EmbR in regulating the transcription of *embCAB* genes by performing quantitative real-time PCR (qRT-PCR) using cDNA obtained from *Rv* and *RvΔembR* strains. We did not observe any discernible difference in the transcript levels of *embCAB* genes, suggesting that EmbR does not regulate this operon’s expression under regular growth conditions (Fig. 3f). Previous studies suggested a link between mutations in *embCAB* and *embR* genes to EMB resistance. Thus, we assessed the impact of *embR* deletion on EMB resistance by determining MIC values for isoniazid (control) and EMB in *Rv*, *RvΔembR*, and *RvΔembR*::*embR* strains. The MIC values obtained for both INH and EMB were comparable in all three strains, emphasizing that EmbR does not play a role in antibiotic resistance to these antibiotics (Fig. 3g). Together, these results suggest that even though EmbR modulates cell morphology, it does not regulate *embCAB* genes and affect EMB susceptibility.

### Deletion of EmbR attenuates intracellular survival of M. tuberculosis.

**FIG 4:**
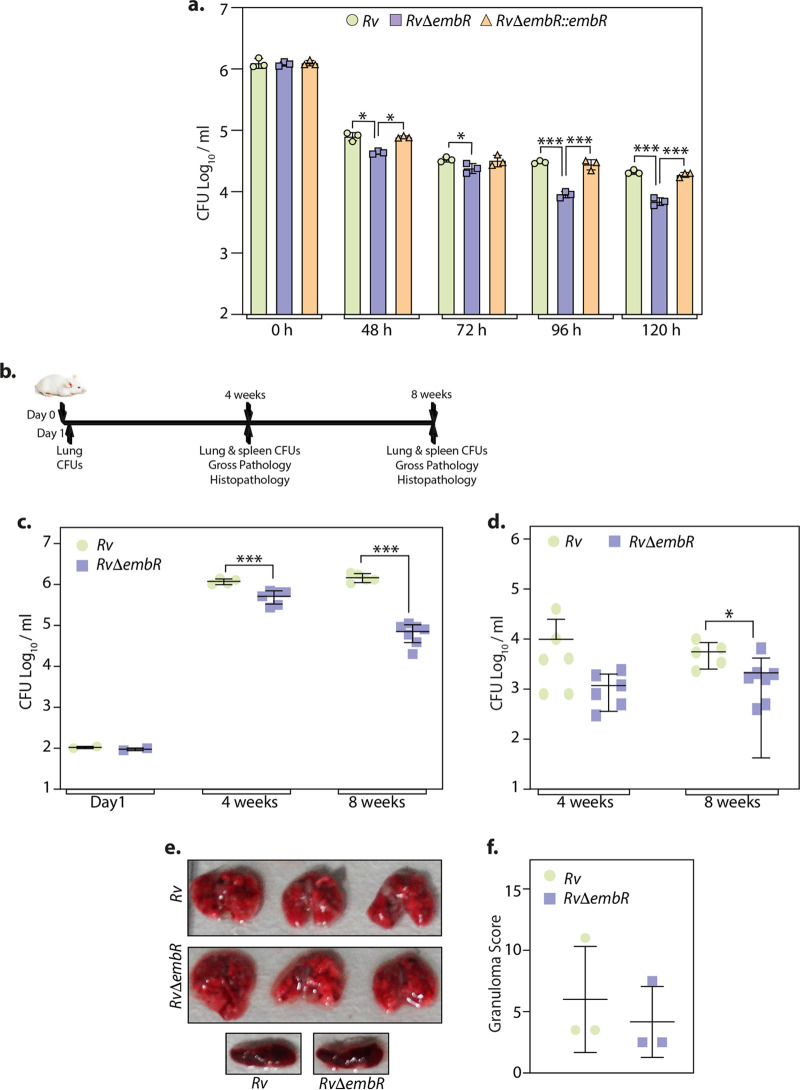
Deletion of EmbR in M. tuberculosis attenuates its ability to survive in the host. (a) Murine peritoneal macrophages isolated from BALB/c mice were infected with *Rv*, *RvΔembR*, and *RvΔembR*::*embR* strains at an MOI of 1:10. At indicated time points, the cells were lysed with 0.05% SDS and bacillary survival was monitored. Data are represented as mean CFU log_10_/mL ± SD and are representative of two independent biological experiments, each performed in triplicates (*n* = 3). (b) Schematic representation of mouse infection experiment. (c) BALB/c mice (*n* = 6) were infected with 100 CFU/mouse *Rv* and *RvΔembR* strains, and the bacterial deposition was determined in the lung homogenates (*n* = 2) at day 1 postinfection (p.i.). Mean log_10_ CFU obtained for *Rv* and *RvΔembR* strains were 6.07 and 5.66 at 4th week and 6.16 and 4.76 at 8th week. (d) Mean log_10_ CFU obtained for *Rv* and *RvΔembR* strains was 3.99 and 3.07 at 4th week and 3.74 and 3.32 at 8th week in infected animals’ spleens. Data are represented as mean CFU log_10_/mL ± SD. *, *P < *0.05; **, *P < *0.005; ***, *P < *0.0005. (e) Lungs and spleen of infected mice depicting gross pathology. (f) Total granuloma score (mean ± SD) in hematoxylin-eosin-stained lung sections of animals infected with M. tuberculosis strains at 8 weeks p.i. Each data point in panels c, d, and f represents data value from one infected animal.

To evaluate the role of *embR* in the *ex vivo* and *in vivo* survival of M. tuberculosis, we performed studies in peritoneal macrophages and a murine infection model. Peritoneal macrophages were infected with *Rv*, *RvΔembR*, and *RvΔembR*::*embR* strains, and CFU were enumerated at various time points postinfection. Compared with *Rv* and *RvΔembR*::*embR* strains, we observed 5-fold reduction in the survival in the *RvΔembR* mutant at 96 and 120 h postinfection (Fig. 4a), which suggested that EmbR promotes mycobacterial survival within the host. To investigate the role of EmbR in the survival of the pathogen *in vivo*, we performed murine infections in mice with *Rv* and *RvΔembR* strains through aerosol routes. The CFU present in the lung and spleen were enumerated 1 day and 4 and 8 weeks postinfection (Fig. 4b). The bacillary load at day 1 suggested efficient and equivalent deposition of all three strains (Fig. 4c). Disease progression was assessed by examining lungs and spleen bacillary load 4 and 8 weeks postinfection. While the bacillary load at day 1 was comparable for *Rv* and *RvΔembR* strains, the mutant showed compromised survival at 4 and 8 weeks (Fig. 4c). Furthermore, we observed a significant reduction in the bacillary load at 8 weeks in the mutant compared with the wild type, indicating that EmbR plays a critical role in the chronic phase of infection. The splenic bacillary load in *RvΔembR* mutant was also observed to be lower than that of the wild type, suggesting reduced dissemination of bacilli in the animals infected with mutant strain (Fig. 4d). Consistent with the above-described observations, we also found a reduced number of granulomas by gross pathology in the lungs of mice infected with *RvΔembR* mutant (Fig. 4e). However, these differences were not reflected in the granuloma score obtained from the histopathological analysis of lung sections, probably due to high variation and possibly fewer replicates (Fig. 4f). Collectively the data strongly support that EmbR may play a role in modulating the survival at later stages of infection, suggesting a correlation with bacterial virulence.

### EmbR regulates the expression of genes involved in virulence, secretion, polyketide synthesis, and hypoxia.

**FIG 5:**
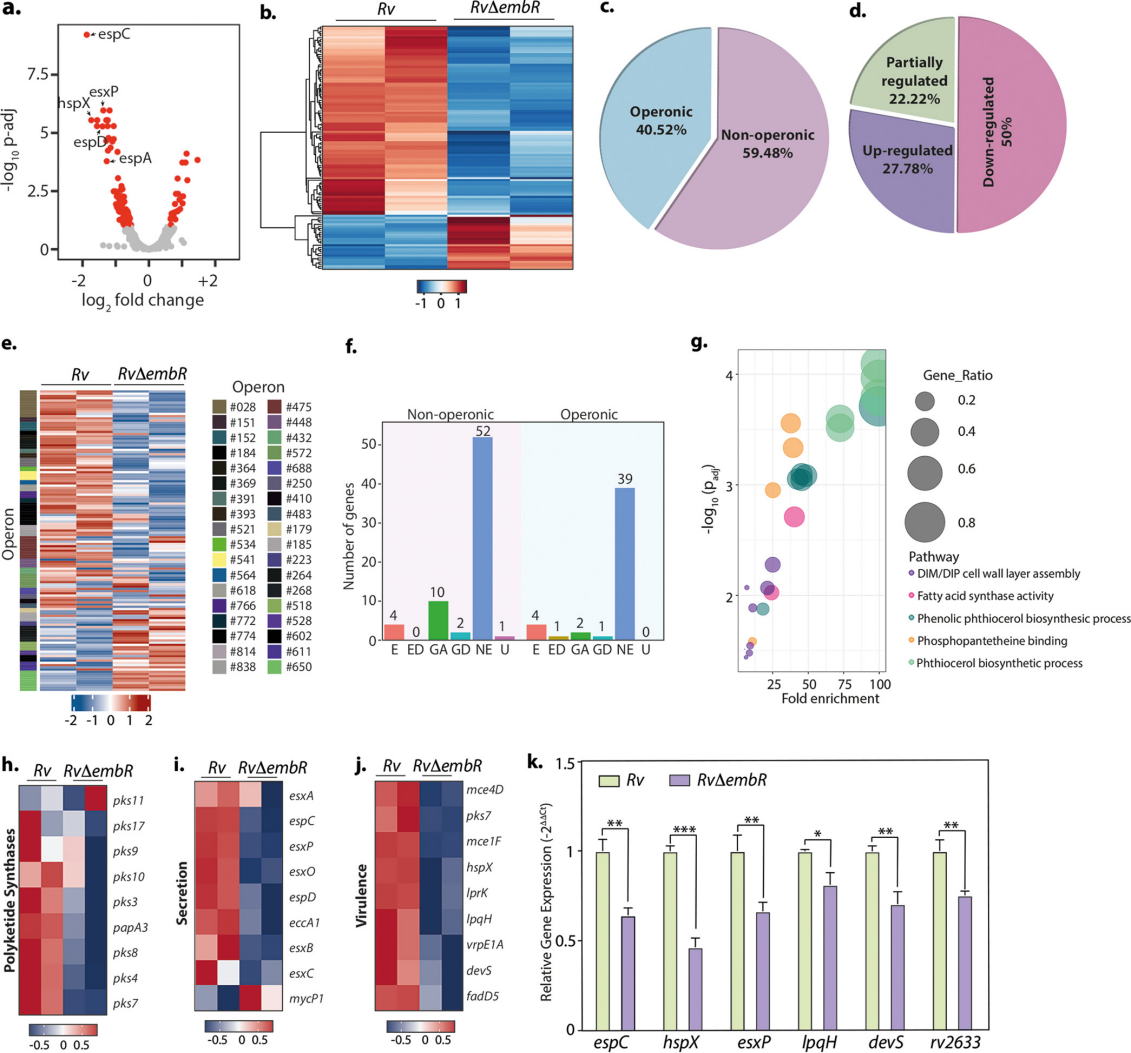
EmbR modulates the expression of various polyketide synthases, virulence factors, and secretory proteins. (a) Volcano plot illustrating differentially expressed genes (DEGs) in *Rv* compared with *RvΔembR* strain. Red spots indicate the genes that show differential expression (*P*adj < 0.1). (b) Heatmaps showing the normalized read counts of DEGs in both biological replicates of *Rv* and *RvΔembR* strains. Color intensity indicates relative up- or downregulation. (c) Pie chart depicting the percentage of DEGs that are operonic and nonoperonic. (d) Pie chart depicting the percentage of operonic DEGs that are fully upregulated, fully downregulated, or partially regulated. (e) Heatmaps showing the normalized read counts of differentially regulated operons along with their numbers in both biological replicates of *Rv* and *RvΔembR* strains. Color intensity indicates relative up- or downregulation. (f) Bar graph demonstrating the number of operonic and nonoperonic DEGs that belong to essential (E), essential domain (ED), growth defect (GD), growth advantage (GA), nonessential (NE), and uncertain (U) categories. (g) The six most significantly enriched Gene Ontology (GO) biological process categories of upregulated DEGs. (h to j) Heat maps showing the normalized read counts in both biological replicates of *Rv* and *RvΔembR* strains of DEGs that are involved in polyketide synthesis (h), genes involved in virulence (i), and genes involved in the secretion pathway (j). Color intensity shows relative up- or downregulation. (k) Selected DEGs obtained from RNA-seq analysis were validated through qRT-PCR. Data were normalized with respect to 16S rRNA and plotted as mean ± SD, representative of two independent biological experiments, each performed in triplicates (*n* = 3). ***, *P* < 0.0005; **, *P* < 0.005; *, *P* < 0.05; not significant, ns.

Thus far, results have negated the prevailing hypothesis on (i) how EmbR transcriptional activity is regulated and (ii) how it modulates the cellular processes. Since EmbR is a transcription factor, we performed a global transcriptomic analysis of *Rv* and *RvΔembR* strains using Illumina-based RNA sequencing from two independent biological replicates. Differential gene expression (DEG) analysis was performed using DESeq2 and is depicted in the volcano plot, where the red dots represent genes that are differentially expressed (1.5-fold change; adjusted *P* value [*P*adj] of <0.1) (Fig. 5a and Table S1a). Heat map generated for both biological replicates for each sample represents a complete set of up- and downregulated genes in *Rv* and *RvΔembR* mutant (Fig. 5b). Deletion of EmbR resulted in a total of 116 DEGs, of which 90 genes were downregulated, indicating EmbR was a plausible transcriptional activator (Table S1a). In consonance with qRT-PCR data presented in Fig. 4c, we did not detect *embCAB* operon genes or those involved in arabinogalactan or lipoarabinomannan biosynthesis among the DEGs. Next, we predicted the operonic arrangement of these 116 DEGs using the Rockhopper tool (27) and found that 47 (40.52%) DEGs are part of 36 predicted operons (Fig. 5c and Table S1b).

10.1128/mbio.03836-21.1 TABLE S1 DEseq and Gene Ontology under normoxic conditions. (a) DEseq2 analysis of *Rv* versus *RvΔembR* strains in normoxic conditions. (b) Operonic arrangement of genes in *Rv*. (c) Gene Ontology analysis of differentially expressed genes under normoxic conditions. Download Table S1, XLSX file, 0.4 MB. Copyright © 2022 Kumar et al. 2022 Kumar et al. https://creativecommons.org/licenses/by/4.0/ This content is distributed under the terms of the Creative Commons Attribution 4.0 International license.

Although not all the genes of these operons met the thresholds set for DEGs, we examined if all the genes in a given operon show a similar trend in the expression level changes. Interestingly, 50% of these operons (18) show decreased transcript levels for all the genes in the operon, while all the genes in 10 operons (27.78%) show increased transcript levels in the mutant (Fig. 5d and e). Next, we investigated how many of these genes are essential for the bacillary survival *in vitro* based on published data (25) and found that among the 116 genes that are differentially expressed, only eight are annotated as essential for *in vitro* growth (Fig. 5f). Interestingly, the six most significantly enriched biological processes that were found among the upregulated genes are related to lipid metabolism (Fig. 5g and Table S1c), explaining altered cellular length and cell wall architecture of the mutant strain. Since we observed that the mutant strain shows attenuated survival *in vivo*, we assessed the expression levels of virulence-associated genes. Indeed, the volcano plot (Fig. 5a) contains several virulence-associated genes that are downregulated in the mutant strain.

Further analysis of the data confirmed that several genes associated with processes required for virulence, such as lipid metabolism and secretion, are downregulated in the mutant strain (Fig. 5h to j). Interestingly, among the virulence factor genes, we found vital hypoxia regulators *devS* and *hspX*, suggesting a role of EmbR in adaptation to hypoxic conditions. Subsequently, we selected six highly downregulated *RvΔembR* mutants associated with hypoxia and virulence to validate the transcriptome sequencing (RNA-seq) data. qRT-PCR was performed to determine the expression profile of *espC*, *hspX*, *esxP*, *lpqH*, *devS*, and *Rv2633* genes in *Rv* and *RvΔembR* mutant (Fig. 5k). The qRT-PCR data were in accordance with the RNA sequencing data. Together, the RNA sequencing data suggest that EmbR is a transcriptional modulator regulating multiple pathways important for virulence.

### EmbR aids in M. tuberculosis survival during hypoxia.

**FIG 6:**
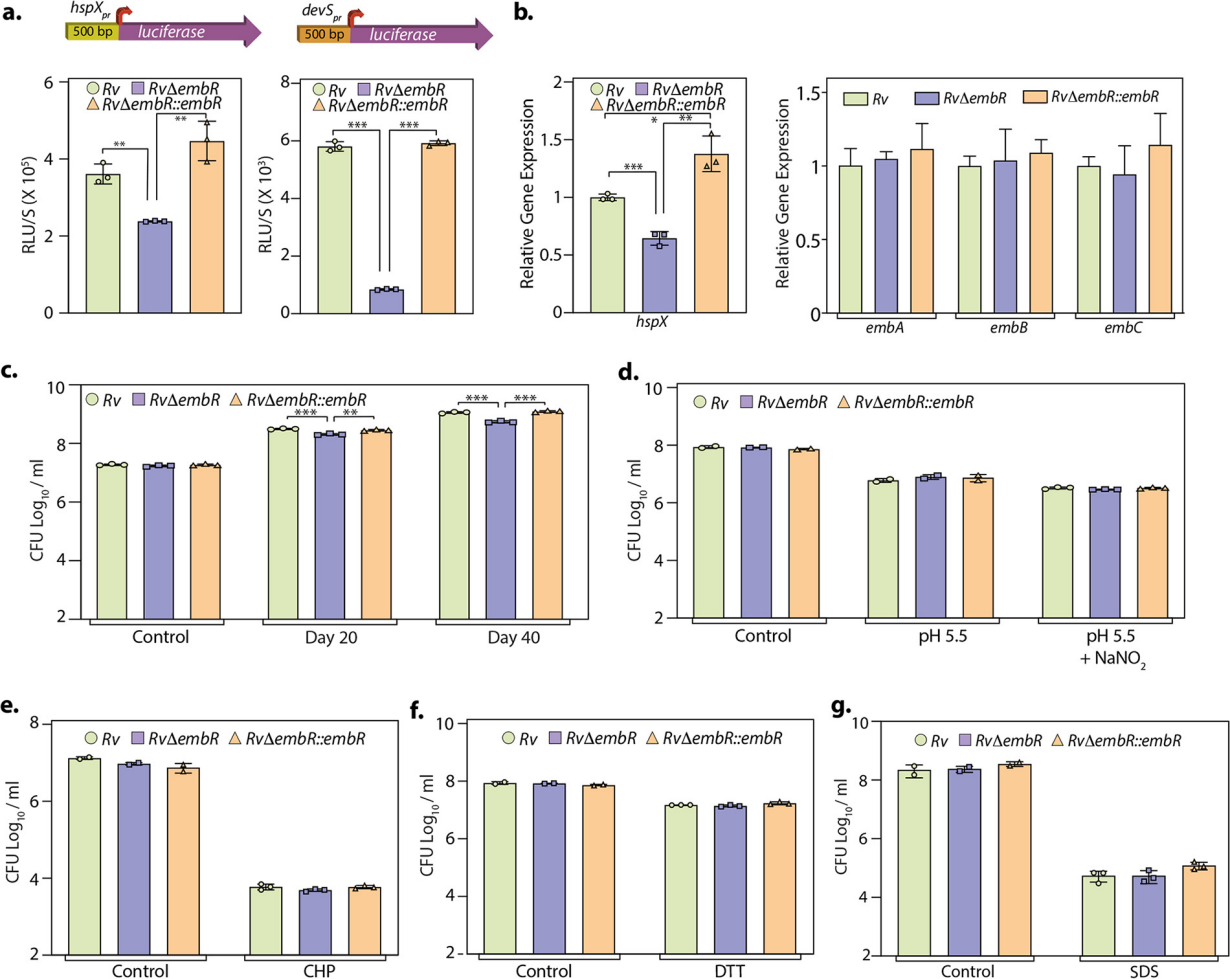
EmbR positively regulates hypoxia-related gene *hspX.* (a) *Rv*, *RvΔembR*, and *RvΔembR*::*embR* strains were electroporated with pSW-hspX_pr_-luciferase and pSW1-devS_pr_-luc construct, and luciferase activity was measured in the WCLs of the transformants. Experiment was performed in triplicates (*n* = 3). (b and c) *Rv*, *RvΔembR*, and *RvΔembR*::*embR* cultures were inoculated at an OD_600_ of ~0.1 and subjected to hypoxic stress in tightly sealed tubes for 20 and 40 days. The data were plotted as means ± SD. The experiment was performed in triplicates (*n* = 3). ***, *P* < 0.0005; **, *P* < 0.005; *, *P* < 0.05. (b) The expression of *hspX*, *embA*, *embB*, and *embC* genes was assessed through qRT-PCR at the 40th day. The data were normalized with respect to 16S rRNA. (c) CFU were enumerated to quantify bacterial survival at indicated time points. (d to g) *Rv*, *RvΔembR*, and *RvΔembR*::*embR* strains were assessed for their susceptibility to *in vitro* stresses. Strains were subjected to nitrosative stress with 3 mM for 2 days (d), oxidative stress with 50 μM CHP for 24 h (e), reductive stress with 1 mM DTT for 24 h (f), and surfactant stress with 0.1% SDS for 3 h (g). CFU were enumerated on 7H11 plates after subjecting cells to indicated stresses. Data are presented as mean CFU log_10_/mL ± SD. Control sample testing in each case was performed in duplicates. Stress samples with nitrosative, oxidative, reductive, or surfactant were performed in triplicates.

Mycobacterial adaptation to hypoxia is thought to have an essential role in disease pathogenesis. During hypoxia, two-component sensor and response regulator *dosS* (*devS*) and *dosR* (*devR*) are upregulated in a coordinated manner (28, 29). *hspX* (*Rv2031*), a dominant antigen required for M. tuberculosis growth and disease pathogenesis *in vivo*, is also known to be induced under hypoxic stress (30). Since both *devS* and *hspX* were significantly downregulated in *RvΔembR* mutant, we speculated that EmbR plays a role in combating hypoxic stress found in the granuloma. To confirm EmbR-mediated regulation of the *hspX* promoter, we performed a luciferase reporter assay, wherein the *hspX* promoter was cloned upstream of the *luciferase* gene. The reporter construct was electroporated into *Rv*, *RvΔembR*, and *RvΔembR*::*embR* strains and luciferase activity was determined in the lysates. Luciferase activity in the lysates of *RvΔembR* mutant was ~1.5-fold lower than activity in the lysates of *Rv* (Fig. 6a). Similarly, *devS* promoter-driven luciferase reading was nearly ~5-fold lower in the *RvΔembR* mutant than *Rv* and *RvΔembR*::*embR* strains (Fig. 6a). Next, we inspected the DNA binding ability of EmbR to biotinylate *hspX* promoter with the help of surface plasmon resonance (SPR). However, we did not observe any significant binding of EmbR to *hspX* promoter under the tested conditions (data not shown), which may be due to the absence of phosphorylation in our *in vitro* study, the requirement of additional interacting partners, such as a sigma factor, or specific *in vivo* conditions. Additionally, it is also possible that EmbR indirectly regulates *hspX* expression, for example, through another activator. Importantly, the expression profile of *hspX* in the *Rv*, *RvΔembR*, and *RvΔembR*::*embR* strains subjected to hypoxic stress suggested a 50% decrease in the expression of *hspX* in *RvΔembR* strain compared with *Rv* (Fig. 6b). Notably, there were no expression changes found in the levels of *embCAB* upon EmbR deletion, even during hypoxic conditions. The luciferase activity and *hspX* expression were higher in the complementation strain than in both *Rv* and *RvΔembR* mutant, which could be due to differences in the expression levels of EmbR (Fig. 6a and b). Subsequently, we examined the impact of *embR* deletion on the survival of pathogens under hypoxic conditions. We observed a 5-fold reduction in the pathogen’s hypoxic survival upon deletion of *embR*, suggesting that EmbR-mediated transcriptional regulation of hypoxic genes is necessary for combating the stress (Fig. 6c). Together, data suggest that EmbR modulates the transcription of multiple virulence-associated genes, including hypoxia-responsive *hspX*, that aid in mycobacterial growth and survival inside the host.

M. tuberculosis encounters a series of stresses within the host *in vivo*, including reactive oxygen and nitrogen intermediates, low pH, surfactant, and oxygen deprivation (20, 31, 32). Next, we set out to determine whether, in addition to hypoxia, EmbR confers a survival advantage to M. tuberculosis even during other host-engendered stresses. *Rv*, *RvΔembR*, and *RvΔembR*::*embR* strains were treated with 3 mM NaNO_2_ (nitrosative), cumene hydroperoxide-CHP (oxidative stress), 1 mM dithiothreitol (DTT) (reductive stress), and 0.1% SDS (surfactant), and the CFU were enumerated at defined time points. Except for hypoxia, none of the stresses had any impact on the viability of *RvΔembR* mutant (Fig. 6d to g), indicating that EmbR plays a specific role in modulating the survival under hypoxic conditions.

### EmbR regulates the expression of multiple essential genes during hypoxia.

**FIG 7:**
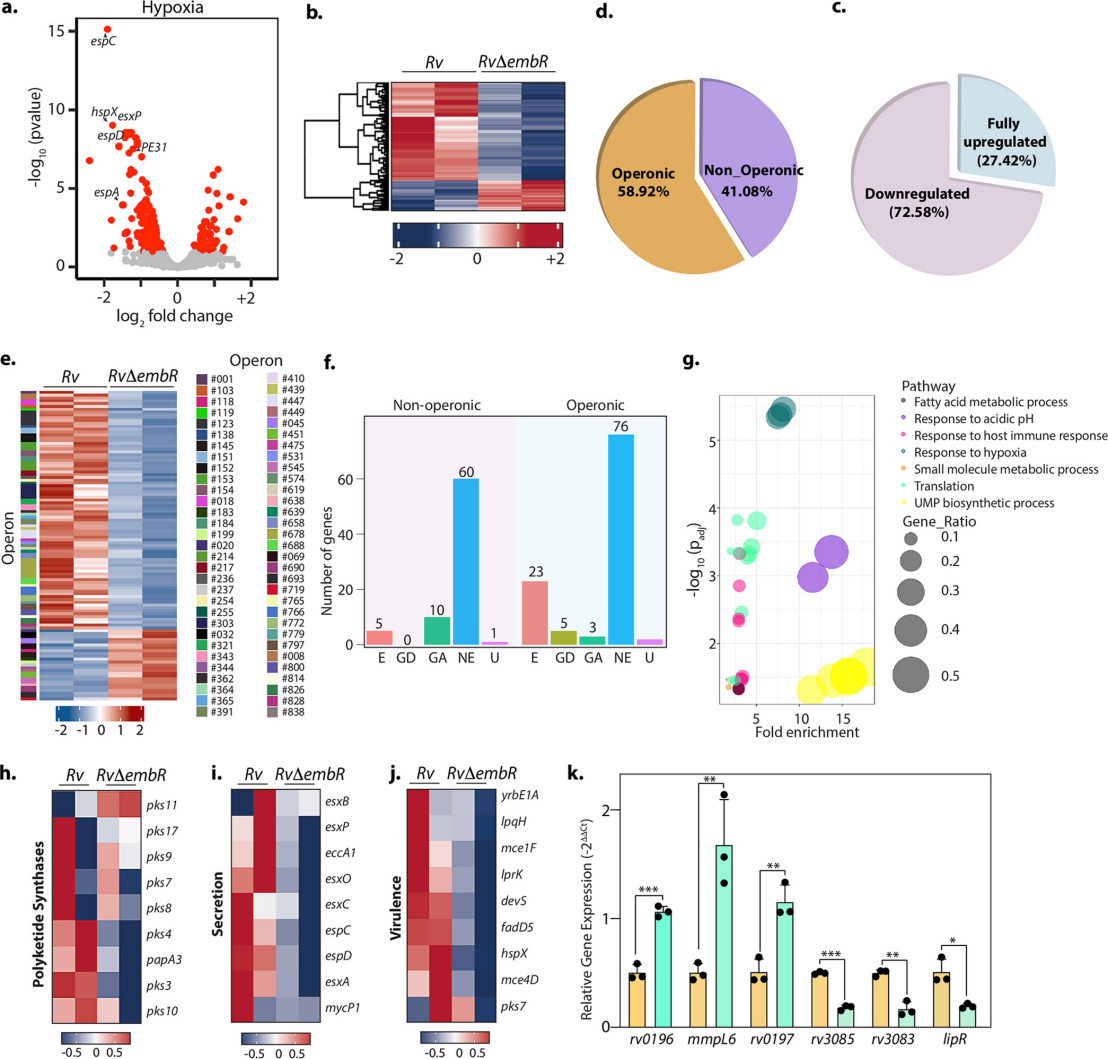
EmbR modulates the expression of various essential genes during hypoxia. (a) Volcano plot illustrating differentially expressed genes (DEGs) in *Rv* compared with *RvΔembR* strain during hypoxia. Red spots indicate the genes that show differential expression (*P*adj < 0.1). (b) Heat maps showing the normalized read counts of DEGs in both biological replicates of *Rv* and *RvΔembR* strains. Color intensity indicates relative up- or downregulation. (c) Pie chart depicting the percentage of DEGs that are operonic and nonoperonic. (d) Pie chart depicting the percentage of operonic DEGs that are fully upregulated and downregulated. Heat maps show the normalized read counts of differentially regulated operons and their number in both biological replicates of *Rv* and *RvΔembR* strains. Color intensity indicates relative up- or downregulation. (f) Bar graph demonstrating a number of operonic and nonoperonic DEGs that belong to essential (E), growth defect (GD), growth advantage (GA), nonessential (NE), and uncertain (U) categories. (g) The six most significantly enriched Gene Ontology (GO) biological process categories of upregulated DEGs. (h to j) Heat maps showing the normalized read counts in both biological replicates of *Rv* and *RvΔembR* DEGs that are involved in polyketide synthesis (h), genes involved in virulence (i), and genes involved in the secretion pathway (j). Color intensity shows relative up- or downregulation. (k) Selected DEGs obtained from RNA-seq analysis were validated through qRT-PCR. Data were normalized with respect to 16S rRNA and plotted as means ± SD. Experiment was performed in triplicates (*n* = 3). ***, *P* < 0.0005; **, *P* < 0.005; *, *P* < 0.05; not significant, ns.

Data presented above suggest that EmbR is specifically necessary for mycobacterial survival during hypoxic stress. Hence, we aimed to evaluate the effect of EmbR deletion on M. tuberculosis transcriptome during hypoxia. Interestingly, EmbR regulates 185 genes during hypoxia compared to 116 genes during regular growth conditions (normoxia) (Fig. 7a and b and Table S2a). As observed during normoxic conditions, the expression of *embCAB* operon genes remains unchanged. Next, we assessed the operonic arrangement of the DEGs and found that 58.92% of DEGs (*P*adj < 0.1) are part of 62 predicted operons (Fig. 7c to e). The majority of the operons that have DEGs were found to be fully downregulated (72.58%) in *RvΔembR* mutant compared to *Rv*, suggesting EmbR is a plausible transcriptional activator even during hypoxia. Importantly, unlike normoxia, where EmbR regulates expression of only 8 *in vitro* essential genes, deletion of EmbR impacts expression levels of 28 *in vitro* essential genes during hypoxia (Fig. 7f). This may explain why deletion of EmbR attenuates mycobacterial survival, specifically during hypoxia. Furthermore, gene ontology analysis suggested genes related to response to acidic pH, host immunity, hypoxia, and fatty acid metabolism are highly enriched among the downregulated genes (Fig. 7g and Table S2b).

10.1128/mbio.03836-21.2 TABLE S2 DEseq and Gene Ontology under hypoxic conditions. (a) DEseq2 analysis of *Rv* versus *RvΔembR* strains in hypoxic conditions. (b) Gene Ontology analysis of differentially expressed genes under hypoxic conditions. Download Table S2, XLSX file, 0.4 MB. Copyright © 2022 Kumar et al. 2022 Kumar et al. https://creativecommons.org/licenses/by/4.0/ This content is distributed under the terms of the Creative Commons Attribution 4.0 International license.

Functional categorization revealed that DEGs during hypoxia included genes involved in polyketide synthesis (Fig. 7h), secretion (Fig. 7i), and virulence (Fig. 7j). To validate the RNA-seq data, we selected three highly upregulated genes and three downregulated *RvΔembR* mutants. qRT-PCR was performed to determine the expression profile of these genes were in accordance with the RNA sequencing data (Fig. 7k).

### EmbR modulates cell wall lipid composition.

In addition to modulation of expression of genes involved in the adaptation to hypoxia, the RNA sequence analysis suggested that EmbR plays a crucial role in the transcription of polyketide synthases and fatty acid synthases during both normoxia and hypoxia (Fig. 5g and 7g). Polyketide synthases are mega-complex proteins that act in a concerted manner with fatty acid synthases to biosynthesize the vast repertoire of functionally and architecturally diverse lipids and glycolipid conjugates present on the M. tuberculosis cell envelope. Notably, several studies have demonstrated the importance of M. tuberculosis lipids as effectors of virulence. The polyketide synthases regulated by EmbR are present in two operons vis-à-vis *pks3-pks4-papA3* and *pks10-pks7-pks8-pks17-pks9-pks11.*

**FIG 8:**
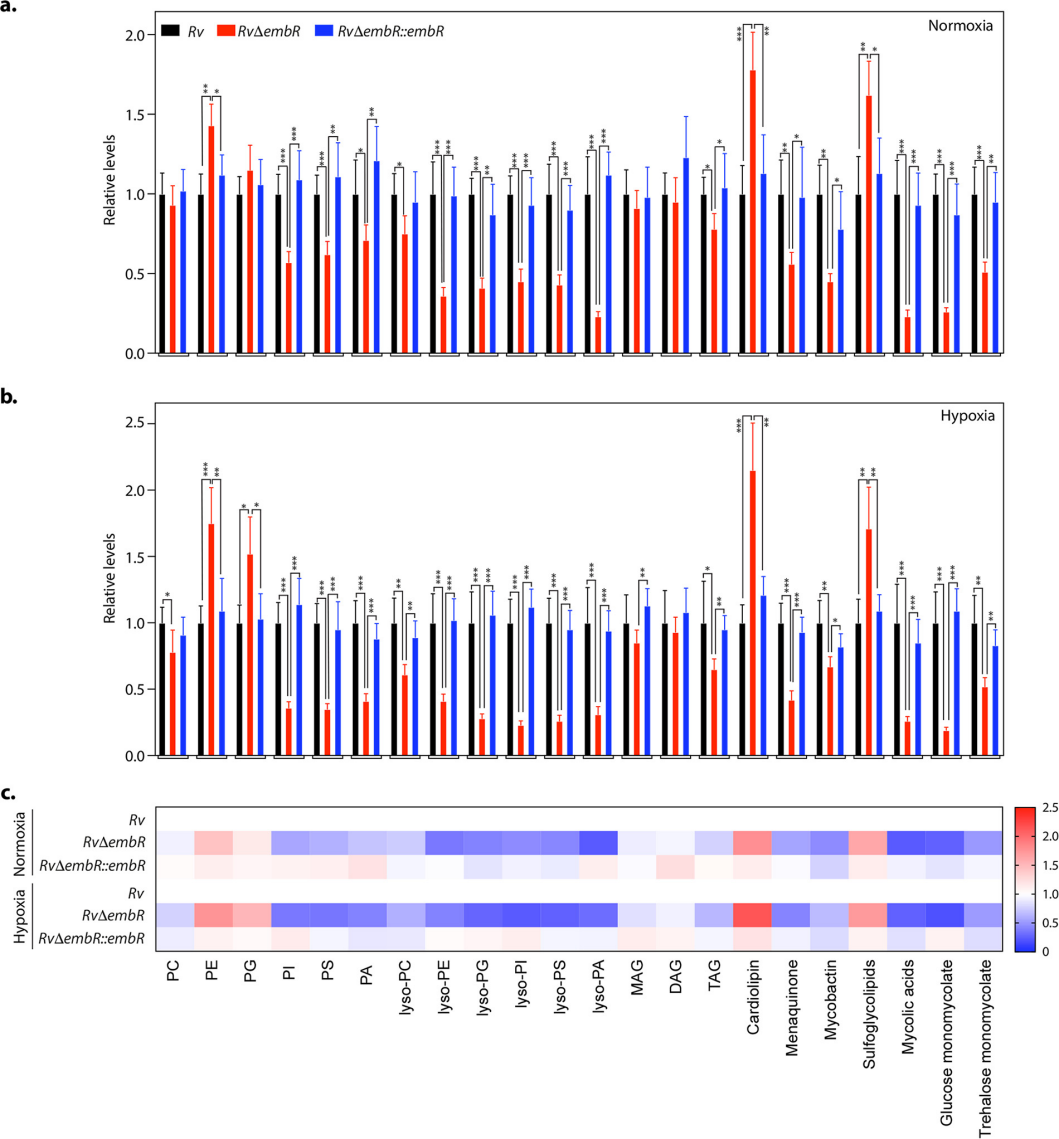
EmbR modulates cell wall lipid composition. (a and b) Relative abundance of the different lipid classes obtained from the total cellular lipids extracted from *Rv*, *RvΔembR*, and *RvΔembR*::*embR* strains grown under normoxic (a) and hypoxic conditions (b). Lipids were analyzed by LC-MS/MS, and resulting abundances were collated as per lipid class, where PC is phosphatidylcholine, PE is phosphatidylethanolamine, PG is phosphatidylglycerol, PI is phosphatidylinositol, PS is phosphatidylserine, PA is phosphatidic acid, MAG is monoacylglycerol, DAG is diacylglycerol, and TAG is triacylglycerol. Data represent means ± SD (*n* = 5). (c) A heat map plot depicting relative changes of various lipid classes in *Rv*, *RvΔembR*, and *RvΔembR*::*embR* strains grown under normoxic and hypoxic conditions.

Next, we aimed to determine whether changes in gene expression of polyketide synthases by EmbR translated into lipid composition changes. To comparatively analyze the lipid profiles, using established protocols (33), we extracted the total cellular lipids from *Rv*, *RvΔembR*, and *RvΔembR*::*embR* strains grown under normoxia and subjected them to an established LC-MS analysis (34, 35) for identification and semiquantitative estimation of the different lipid classes. The abundances of different lipid classes for the *RvΔembR* and *RvΔembR*::*embR* strain groups were normalized to the *Rv* group to yield relative abundances for individual groups. As represented in Fig. 8a, the deletion of *embR* resulted in significant alterations in the overall lipid content of M. tuberculosis (*RvΔembR* strain). These changes in the lipid profiles were restored to almost wild-type levels upon complementation with *embR* (*RvΔembR*::*embR* strain). Specifically, we found that the cellular levels of several phospholipids (PI, PS, and PA), all lysophospholipids, and M. tuberculosis-specific lipids (menaquinone, mycobactin, mycolic acid, glucose monomycolate, and trehalose monomycolate) were most notably reduced upon *embR* deletion (Fig. 8a). Concomitant to these alterations, we also found that deletion of *embR* resulted in an accumulation of some cellular phospholipids (PE, PG, and cardiolipins) and sulfoglycolipids in the *RvΔembR* strain (Fig. 8a). Next, we tested whether EmbR also modulates the lipid profile of M. tuberculosis during hypoxia. Toward this end, *Rv*, *RvΔembR*, and *RvΔembR*::*embR* strains were subjected to hypoxia, and lipidome was measured as described earlier. We found from this lipidomics analysis that while the trend in the lipid alterations remained the same between the normoxic and hypoxic samples, the EmbR-mediated changes for the different lipid classes were more pronounced during hypoxia (Fig. 8a to c and Table S3). Altogether, the lipidomics data suggest that EmbR plays a crucial role in the biosynthesis of various lipids during normoxia and hypoxia.

10.1128/mbio.03836-21.3 TABLE S3 Lipidome analysis under normoxic and hypoxic conditions. Relative values of various lipid classes in *Rv*, *RvΔembR*, and *RvΔembR*::*embR* strains during normoxic and hypoxic conditions. Download Table S3, XLSX file, 0.01 MB. Copyright © 2022 Kumar et al. 2022 Kumar et al. https://creativecommons.org/licenses/by/4.0/ This content is distributed under the terms of the Creative Commons Attribution 4.0 International license.

### EmbR is phosphorylated *in vivo* in a condition-specific manner.

**FIG 9:**
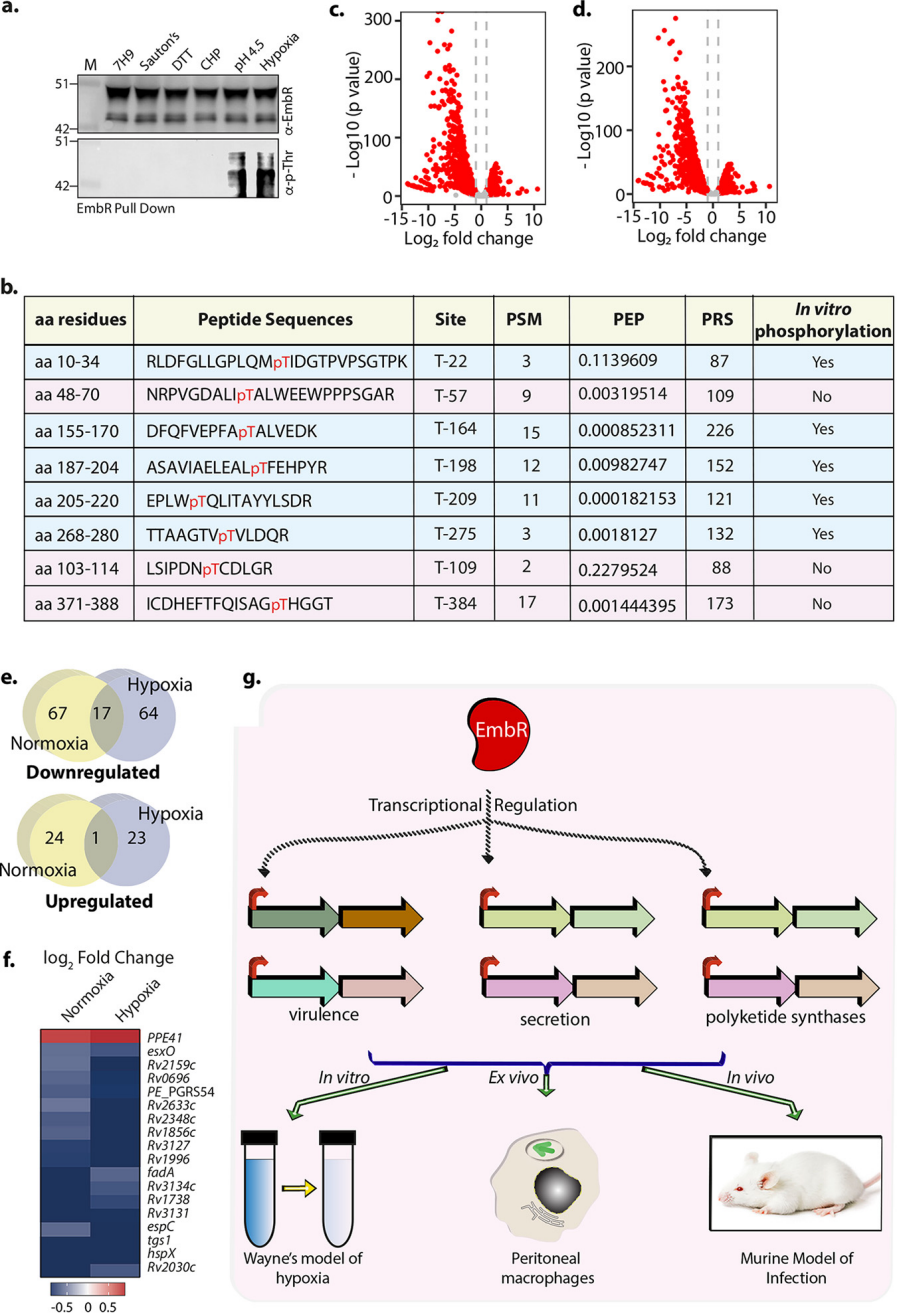
EmbR is phosphorylated *in vivo* in a condition-specific manner. (a) Endogenous EmbR was immunoprecipitated from whole-cell lysates of *Rv* grown under indicated conditions. The immunoprecipitated samples were probed with α-EmbR and α-p-Thr antibodies. (b) Table summarizing eight target phosphorylation sites on EmbR under hypoxic conditions using a mass spectrometer. (c) Volcano plot illustrating differentially expressed genes (DEGs) in *Rv* grown under hypoxic conditions compared with normoxic conditions. Red spots indicate the genes that show differential expression (*P*adj < 0.1). (d) Volcano plot illustrating differentially expressed genes (DEGs) in *RvΔembR* strain grown under hypoxic conditions compared with normoxic conditions. Red spots indicate the genes that show differential expression (*P*adj < 0.1). (e) Venn diagram illustrating the common set of downregulated and upregulated genes under normoxic conditions and hypoxic conditions (log_2_ fold change of less than −0.6). (f) Heat map depicting genes that EmbR regulates under both normoxic and hypoxic conditions. (g) Model depicting EmbR-mediated transcriptional regulation of genes primarily involved in virulence, secretion, and polyketide synthesis. The study establishes the importance of EmbR using *in vitro* hypoxia, *ex vivo* peritoneal macrophage infection, and *in vivo* murine infection model.

Based on the results presented above, we conclude that (i) EmbR modulates M. tuberculosis cell wall composition through altered expression of polyketide synthases and fatty acid synthases; (ii) EmbR modulates key hypoxia-related genes and confers a survival advantage to M. tuberculosis during hypoxia; and (iii) RNA-seq and lipidomics analysis suggested that the extent of EmbR-mediated transcriptional and lipid changes are more pronounced during hypoxic conditions. Since *in vitro* phosphorylation of EmbR is known to upregulate DNA binding ability (19), we speculated that *in vivo* EmbR phosphorylation is context specific. Toward this end, *Rv* was grown in 7H9 and either left untreated or subjected to various stresses, such as nutrient-limiting (Sauton’s), reductive (DTT), oxidative (CHP), acidic (pH 4.5), and hypoxic. Endogenous EmbR protein was immunoprecipitated and probed with α-EmbR and α-p-Thr antibodies (Fig. 8a). In line with the previous results (Fig. 1g), we did not detect any phosphorylation under normal growth conditions. Moreover, phosphorylation could not be detected under nutrient-limiting, reductive, and oxidative conditions. Interestingly, we observed robust phosphorylation of EmbR under acidic and hypoxic conditions (Fig. 9a). Next, we identified the target phosphorylation sites on EmbR under hypoxic conditions using previously described LC-MS analysis. We identified eight phosphorylation sites on EmbR (Fig. 9b and Fig. S3), namely, T22, T57, T109, T164, T189, T209, T275, and T384. All the sites that were identified *in vitro* (Fig. 1f) were detected *in vivo* (Fig. 9b). Importantly, we identified three additional target phosphorylation sites, T57, T109, and T384, specifically under *in vivo* hypoxic conditions.

10.1128/mbio.03836-21.7 FIG S2 MS/MS spectra of PknH target EmbR phosphopeptides. Download FIG S2, TIF file, 1.1 MB. Copyright © 2022 Kumar et al. 2022 Kumar et al. https://creativecommons.org/licenses/by/4.0/ This content is distributed under the terms of the Creative Commons Attribution 4.0 International license.

10.1128/mbio.03836-21.8 FIG S3 MS/MS spectra of EmbR phosphopeptides found during hypoxic growth conditions in *Rv*. Download FIG S3, TIF file, 1.0 MB. Copyright © 2022 Kumar et al. 2022 Kumar et al. https://creativecommons.org/licenses/by/4.0/ This content is distributed under the terms of the Creative Commons Attribution 4.0 International license.

To further investigate the functional relevance of EmbR phosphorylation, we compared the RNA-seq data from hypoxia and normoxia. When subjected to hypoxia, both strains exhibited differential expression of several hundred genes, 2,795 for wild type and 2,195 for *RvΔembR* mutant (Fig. 9c and d and Table S4a and b). To better understand the precise role of EmbR in gene expression associated with hypoxia, we utilized two approaches. First, we compared the genes that are regulated by EmbR in normoxia and hypoxia. Among the downregulated genes (1.5-fold change and *P*adj < 0.1), we found 18 mRNAs had reduced levels under both conditions, indicating that they are regulated by EmbR irrespective of the growth condition (Fig. 9e and f). Only one of the genes on the upregulated gene lists was shared by both conditions (Fig. 9e and f). Interestingly, the fold change is significantly pronounced for 3 out of these 18 DEGs during hypoxia (Table S5a). Next, we asked what fraction of hypoxia-related genes is regulated by EmbR under normoxic and hypoxic conditions. Out of 68 genes that are identified as hypoxia-associated genes in PantherDB, we found that 18 are differentially expressed in *RvΔembR* mutant under hypoxic conditions (Table S5b and c). On the other hand, only 4 of these are differentially expressed in these cells under normoxic conditions. These results indicate that EmbR regulates different subsets of genes under different conditions with some overlap between conditions (Fig. 9e and f). Collectively, these results suggest context-dependent modulation of EmbR activity through phosphorylation.

10.1128/mbio.03836-21.4 TABLE S4 (a) DEseq2 analysis of *Rv* under hypoxic conditions versus normoxic conditions. (b) DEseq2 analysis of *RvΔembR* strain under hypoxic conditions versus normoxic conditions. Download Table S4, XLSX file, 0.7 MB. Copyright © 2022 Kumar et al. 2022 Kumar et al. https://creativecommons.org/licenses/by/4.0/ This content is distributed under the terms of the Creative Commons Attribution 4.0 International license.

10.1128/mbio.03836-21.5 TABLE S5 (a) Statistical analysis for common DEGs in *Rv* versus *RvΔembR* strains under both normoxic and hypoxic conditions. (b) List of common DEGs in *Rv* versus *RvΔembR* strains under both normoxic and hypoxic conditions. (c) Gene ontology analysis of hypoxia associated genes. Download Table S5, XLSX file, 0.02 MB. Copyright © 2022 Kumar et al. 2022 Kumar et al. https://creativecommons.org/licenses/by/4.0/ This content is distributed under the terms of the Creative Commons Attribution 4.0 International license.

## DISCUSSION

SARP family of transcription factors has been associated with regulating gene expression during different host-induced stress conditions and upon antibiotic treatment. One such highly conserved TF belonging to this family in M. tuberculosis is EmbR (16). Based on *in vitro* kinase assays and gel shift assays, PknH is reported to phosphorylate EmbR, which in turn regulates its DNA binding and expression of *embCAB* operon (Fig. 1b) (19–21). However, to date, the function of EmbR *in vivo* has not been evaluated. In this study, we generated a gene replacement mutant (Fig. 1) of *embR* in M. tuberculosis and analyzed its impact on M. tuberculosis growth and survival *in vitro*, *ex vivo*, and *in vivo*.

Protein phosphorylation of specific amino acid residues like serine/threonine or tyrosine is one of the most common regulatory mechanisms in prokaryotes and eukaryotes. M. tuberculosis encodes 11 STPKs that regulate its pathogenicity under various environmental stresses (36). Membrane-bound kinases play a critical role in communicating the external stimuli to the bacterium. EmbR is phosphorylated by multiple STPKs, including PknA, PknB, PknE, PknF, and PknH, *in vitro* (18–22). However, these studies did not identify the target residues on EmbR, whether EmbR is phosphorylated in M. tuberculosis, and its functional implication. Our kinase experiments in the surrogate host E. coli showed PknB, PknD, and PknH robustly phosphorylate EmbR. With the help of mass spectrometry, we identified five target sites for PknB and PknH, with T22 and T275 being common for both kinases. However, despite our efforts using both anti-pThr antibodies and LC-MS analysis, we could not establish that EmbR is phosphorylated in M. tuberculosis during normal conditions. These results are contrary to those obtained by Sharma et al., where, using the anti-pThr antibody, the authors demonstrated the phosphorylation of EmbR in Mycobacterium smegmatis (19). These differences could be due to differences in the host organism used or the fact that we have not overexpressed EmbR in the cell. In a recent high-throughput phosphoproteomics study, one phosphopeptide corresponding to EmbR was identified (37). The absence of phosphopeptides in our targeted LC-MS analysis for EmbR could be because we did not enrich phosphopeptides before this analysis or specific growth and environmental conditions. Nevertheless, our results agree with multiple high-throughput phosphoproteomic studies where phosphopeptides corresponding to EmbR have not been identified under normal growth conditions (38, 39). Thus, it is likely that even if EmbR is phosphorylated *in vivo*, the stoichiometry of phosphorylation is very low, suggesting the phosphorylation is not a major regulatory mechanism under regular growth conditions (Fig. 1).

Interestingly, although the deletion of EmbR was found to be nonessential for the *in vitro* growth of M. tuberculosis (Fig. 2), the deletion resulted in the attenuated survival of bacteria inside host peritoneal macrophages (Fig. 4). Concordantly, murine infection experiments reiterated the importance of EmbR in bacterial survival inside the host with no drastic change in the infected organelles’ histopathology (Fig. 4). These observations demonstrate that EmbR deletion compromises the survival of M. tuberculosis inside the host at later time points, suggesting a role in the chronic phase of M. tuberculosis infection. The rigid mycobacterial cell wall undergoes refurbishment inside the host and acts as one of the innate defense mechanisms against host-induced stresses (40). The thick cell wall acts as an impermeable barrier to most antibiotics. Based on *in vitro* DNA binding experiments and PknH overexpression in M. smegmatis, EmbR has been suggested to regulate *embCAB* operon that has genes involved in arabinogalactan biosynthesis (19). The binding of EmbR to the *embC*, *embA*, and *embB* promoter is expected to modulate its transcriptional levels, affecting the cellular morphology and cell wall architecture. Concordantly, we found significant changes in the cell length, width, and cell wall ultrastructure. However, contrary to the prevailing hypothesis, expression levels of *embCAB* were unchanged in *RvΔembR* mutant (Fig. 3, 5, and 7). Mutations in *embCAB* operon (specifically *embB*) have been associated with EMB resistance in various clinical isolates from different countries (41–45). In line with the qRT-PCR data of *embCAB* presented in Fig. 3f, we did not observe any changes in MIC of EMB upon *embR* deletion (Fig. 3). Collectively, our results negate the proposed hypothesis of regulation of EmbR by phosphorylation and its functional association with *embCAB* genes under normal growth conditions.

Mycobacterial adaptation to different environmental stimuli during infection is key to survival in the host macrophages’ dynamic microenvironment (32, 46). Adaptation to low-oxygen tension found inside the granuloma is thought to be a foremost determinant (31). However, the current understanding regarding the signals and factors that are associated with latency and reactivation is rather limited. During latency, hypoxia and nitric oxide are sensed by DosS (DevS) and DosT (DevT) kinases, which in turn enables bacterial adaptation by transcribing a set of genes through cognate response regulator DosR (DevR) (28, 29, 47). HspX is an antigen regulated by DevR and is the most abundant upregulated protein during hypoxia (30). At later time points during hypoxia, enduring hypoxia response (EHR) is mediated by different transcriptional regulators (31, 48). Transcriptome profile of *embR* deletion strain revealed DEG mainly involved in virulence, secretion, and polyketide synthesis pathways, which was further confirmed by qRT-PCRs (Fig. 5). Furthermore, we observed hypoxia-related gene downregulation, such as *hspX* and *devS* in *RvΔembR* mutant. Thus, based on transcriptome profiling, we speculated that EmbR mediates adaptation to hypoxia by transcriptional modulation of *hspX* and *devS*. In accordance with this, we observed specific upregulation of *hspX* promoter-driven luciferase activity in *Rv* and *RvΔembR*::*embR* mutant. Importantly, we found that EmbR modulates *hspX* transcripts levels. Significantly, the deletion of EmbR attenuates mycobacterial survival, specifically during hypoxia (Fig. 6). Furthermore, we found EmbR regulates the expression of 185 genes during hypoxia (Fig. 7).

The rather unusual cell wall of M. tuberculosis comprises three core layers made up of peptidoglycan, mycolic acids, and arabinogalactan. Several free lipids, phosphatidylinositol mannosides, lipomannans, and lipoarabinomannans, intersperse between these core cell wall layers. In addition to maintaining cell wall and shape, the diverse repertoire of mycobacterial lipids also plays a key role in cellular signaling, initiation of the innate response, and antibiotic resistance. We found that EmbR positively regulates the expression of polyketide synthases and fatty acid synthases, enzymes that biosynthesize mycobacterial lipids. Consequently, 18 out of 22 complex mycobacterial lipids analyzed by LC-MS/MS were found to be downregulated in *RvΔembR* mutant compared with *Rv* and *RvΔembR*::*embR* mutant (Fig. 8). The degree of differences in lipid abundances between *Rv*, *RvΔembR* mutant, and *RvΔembR*::*embR* mutant was intensified during hypoxia. Interestingly, the mycobacterial cell wall undergoes cell wall remodeling to adapt to hypoxic stress. Hence, we speculate that reduced survival of *RvΔembR* mutant during hypoxia is due to EmbR’s ability to modulate the expression of polyketide synthases, fatty acid synthases, and essential hypoxia-associated genes such as *devS* and *hspX*. Sharma et al. showed that *in vitro* phosphorylation of EmbR by PknH increased phosphorylation of EmbR is a positive modulator of its DNA binding ability for *embC*, *embA*, and *embB* promoter regions (19). While EmbR is not phosphorylated under regular growth conditions (Fig. 1), it is specifically phosphorylated under acidic and hypoxic conditions (Fig. 9). However, this was not reflected *in vivo* in reduced transcript levels of *embCAB* in EmbR mutant under normoxic or hypoxic conditions. The disparity in the observations between *in vitro* and *in vivo* data may be due to the additional factors/interactions of EmbR or DNA::p-EmbR stoichiometry.

Furthermore, EmbR-dependent lipidome regulation is more pronounced under hypoxic conditions (Fig. 8). Hence, it is conceivable that EmbR phosphorylation enhances its DNA binding under hypoxic conditions, altering the expression of its target hypoxia-associated genes. Concordantly, we found expression of 185 genes was regulated by EmbR during hypoxia, compared to 116 DEGs during normoxia (Fig. 5 and 7). While a subset of EmbR regulons is common during either of the tested conditions, the majority of the genes (90.81%) are regulated in a condition-specific manner (Fig. 9). Together, our study establishes a crucial role of EmbR as a transcriptional modulator of genes belonging to multiple pathways, *viz.*, virulence, secretion, or polyketide synthesis, that aid in mycobacterial survival during hypoxia and within the host (Fig. 9f).

## MATERIALS AND METHODS

### Bacterial strains and growth conditions.

Table 1 describes the bacterial strains and constructs used in the study. Escherichia coli DH5α (Invitrogen) and *E. coli* BL21(DE3) codon plus (Stratagene) strains were used for cloning and protein purification, respectively. 7H9 medium supplemented with 10% ADC (NaCl, dextrose, bovine serum albumin, and catalase), 0.2% glycerol, and 0.1% Tween 80 was used for the liquid growth of M. tuberculosis strains. 7H11 agar with 10% OADC (ADC along with oleic acid) and 0.2% glycerol was used for M. tuberculosis strain growth on plates. E. coli transformants were selected on ampicillin (100 μg/mL)–kanamycin (50 μg/mL)–hygromycin (150 μg/mL)–chloramphenicol (34 μg/mL) or apramycin (30 μg/mL). M. tuberculosis recombinants were selected on kanamycin (25 μg/mL)–hygromycin (100 μg/mL)–chloramphenicol (40 μg/mL) or apramycin (30 μg/mL). Medium components were from BD Difco, Sigma-Aldrich, and Hi-Media. Molecular grade reagents were procured from Merck, Ameresco, or Sigma; Luciferase reporter assay kit was from Promega (number E1500); restriction-modification enzymes were from NEB; and SEM chemicals were from Electron Microscopy Sciences. Oligonucleotides (Table 2) were procured from Sigma.

**TABLE 1:** Constructs generated and strains used in this study

Construct	Description^*a*^	Antibiotic resistance	Source or reference
pET-Duet-MBP	MBP tag cloned into NdeI-EcoRV of MCSII	Amp^r^	62
pET-Duet-MBP-EmbR	pET-Duet-MBP modified by inserting EmbR into the unique HindIII in MCSI	Amp^r^	This study
pET-Duet-MBP-PknA-EmbR	pET-Duet-MBP-PknA modified by inserting EmbR into the unique HindIII in MCSI	Amp^r^	This study
pET-Duet-MBP-PknB-EmbR	pET-Duet-MBP-PknB modified by inserting EmbR into the unique HindIII in MCSI	Amp^r^	This study
pET-Duet-MBP-PknD-KD-EmbR	pET-Duet-MBP-PknD-KD modified by EmbR into the unique HindIII in MCSI	Amp^r^	This study
pET-Duet-MBP-PknE-KD-EmbR	pET-Duet-MBP-PknE-KD modified by EmbR into the unique HindIII in MCSI	Amp^r^	This study
pET-Duet-MBP-PknF-KD-EmbR	pET-Duet-MBP-PknF-KD modified by EmbR into the unique HindIII in MCSI	Amp^r^	This study
pET-Duet-MBP-PknG-EmbR	pET-Duet-MBP-PknG modified by inserting EmbR into the unique HindIII in MCSI	Amp^r^	This study
pET-Duet-MBP-PknH-KD-EmbR	pET-Duet-MBP-PknH modified by EmbR into the unique HindIII in MCSI	Amp^r^	This study
pET-Duet-MBP-PknI-KD-EmbR	pET-Duet-MBP-PknI-KD modified by EmbR into the unique HindIII in MCSI	Amp^r^	This study
pF-F-embR	*embR* ORF cloned into NdeI-HindIII sites in pFICTO (65) vector	Kan^r^	This study
pF-embR-HA	*embR* ORF* *+* *500-bp upstream region cloned into ScaI-HindIII sites in pFICTO vector	Chl^r^	This study
pSW-luc	*Luciferase* ORF cloned into the NdeI-HindIII sites in the MCS of pSW vector (unpublished)	Apra^r^	Unpublished data
pSW-luc-hspX	*hspX* promoter region cloned into unique ScaI-NdeI sites of pSW-luc	Apra^r^	This study
pNit-pknB	*pknB* ORF cloned into NdeI-HindIII sites in MCS of pNit1 vector	Kan^r^	This study
pNit-pknH	*pknH* ORF cloned into NdeI-HindIII sites in MCS of pNit1 vector	Kan^r^	This study
pNit-pknF	*pknF* ORF cloned into NdeI-HindIII sites in MCS of pNit1 vector	Kan^r^	This study
DH5α	*E. coli* strain used for cloning experiments		Invitrogen
BL21 (DE3) Codon Plus	*E. coli* strain used for protein expression experiments		Stratagene
*H37Rv (Rv)*	Virulent laboratory strain of M. tuberculosis used as wild type		ATCC
*RvΔembR*	EmbR deletion mutant	Hyg^r^	This study
*RvΔembR*::*embR*	*RvΔembR* mutant electroporated with pF-embR-HA harboring carboxy-terminal 3× HA tag	Hyg^r^, Chl^r^	This study
*RvΔembR*::*FembR*	*RvΔembR* mutant electroporated with pF-F-embR containing 3× FLAG tag at the N terminus	Hyg^r^, Chl^r^	This study
*RvΔembR*::*FembR-pknB*	*RvΔembR*::*FembR* electroporated with pNit-pknB	Hyg^r^, Chl^r^, Kan^r^	This study
*RvΔembR*::*FembR-pknH*	*RvΔembR*::*FembR* electroporated with pNit-pknH	Hyg^r^, Chl^r^, Kan^r^	This study
*RvΔembR*::*FembR-pknF*	*RvΔembR*::*FembR* electroporated with pNit-pknF	Hyg^r^, Chl^r^, Kan^r^	This study

a ORF, open reading frame.

**TABLE 2:** List of primers used in this study

Reference no.	Description	Sequence (5′–3′)
STL261	*embR*-AES 5′ flank forward harboring AlwNI and SnaBI sites	CACCTTTTCAGAAACTGTACGTACACGCCGTGATCGTCAGCATC
STL262	*embR*-AES 5′ flank reverse harboring AlwNI site	TTTTTTTTCAGTTCCTGTACGCAGATTAGACACGTAGG
STL263	*embR*-AES 3′ flank forward harboring AlwNI site	CACCTTTTCAGAGACTGTGCATGTGCAGCACGAGCGAA
STL264	*embR*-AES 3′ flank forward harboring AlwNI and SnaBI sites	TTTTTTTTCAGCTTCTGTACGTAAAGTCGACCAGATAGGCAAAG
STL181	*Hyg* end sequence forward for *embR* mutant screening	CGATCCGGAGGAACTGGCGCA
VKN415	*pknH* end reverse for *embR* mutant screening harboring NotI site	AGCTGCGGCCGCTCATTCCTTGTTGACTTTGTC
STL53	*pknH* middle reverse for *embR* mutant screening harboring HindIII site	AGCTAAGCTTCTATGCGGGTGGCTGCCGAGGAGC
VKN277	*embR* middle forward for *embR* mutant screening harboring DraIII site	TTTTTTTTCACAAAGTGGTCAGCAACCGCTGGATGCC
VK98	*embR* forward harboring NdeI site	CACCCATATGGCTGGTAGCGCGACAGTG
VK99	*embR* reverse harboring HindIII site	AAGCTTGATCTACGTGCCGCCATGCGT
BSL31	*embR* promoter forward (500 bp upstream) harboring ScaI site	CACCAGTACTAAAACGGCATTGTTCACCTC
BSL32	*embR* reverse without stop codon harboring HindIII site	AGCTAAGCTTCGTGCCGCCATGCGTCCC
STL265	*embR* forward for pDuet harboring HindIII site	CACCTAAAGCTTATGGCTGGTAGCGCGACAG
STL266	*embR* reverse for pDuet harboring HindIII and AfII sites	TTTCTTAAGCTTCTACGTGCCGCCATGCGTC
BSL400	*hspX* promoter forward harboring ScaI site	CCCAGTACTAAGTCAATTGACGCCAGA
BSL401	*hspX* promoter reverse harboring NdeI site	CCCCATATGATGCCTCCTAATCGATGG
VK900	16S rRNA qRT forward primer	ACGAACAACGCGACAAACC
VK901	16S rRNA qRT reverse primer	CCAGCAGCCGCGGTAA
BSL29	*embC* forward qRT primer	AATTGTCCAGTCCCCGTTG
BSL30	*embC* reverse qRT primer	CAAAGCCTGTAGGTTAGACCG
STL1169	*embA* forward qRT primer	TGGTTCTACGTCGGCAACTA
STL1170	*embA* reverse qRT primer	GTCTTTGACTTCGGTGTGCC
STL1173	*embB* forward qRT primer	TATACGGAGAGCAGCCCAAG
STL1174	*embB* reverse qRT primer	GCCCACATATTCCTGCAGTG
BSL375	*hspX* forward qRT primer	ACATTATGGTCCGCGATGG
BSL376	*hspX* reverse qRT primer	ACCGACACAGTAAGAATGCC
BSL377	*esxP* forward qRT primer	GGCAACACGTTTTATGACGG
BSL378	*esxP* reverse qRT primer	CATGGTGTCTAGCGAGGTC
BSL379	*Rv2633* forward qRT primer	ACATTCACTTCCGCATCGAG
BSL380	*Rv2633* reverse qRT primer	GTTCCACTCTTCTTCATACCCG
BSL381	*espC* forward qRT primer	ATGTGTACTTGACTGCCCAC
BSL382	*espC* reverse qRT primer	CCTCGCTATATATCTTCGCCG
BSL383	*lpqH* forward qRT primer	GAGGTGAAGTCCGTTGGG
BSL384	*lpqH* reverse qRT primer	GGTCCCAGTGATCTTGTAGTG
BSL385	*devS* forward qRT primer	TCGAAGATCCCAAACCGTTAC
BSL386	*devS* reverse qRT primer	AGAGTGCCGAACGATTCATC

### Generation of *embR* deletion mutant in M. tuberculosis.

Upstream and downstream regions (~1,000 bp each), including ~200 bp of M. tuberculosis *embR* loci, were amplified from M. tuberculosis genomic DNA using Phu DNA polymerase. Amplicons were digested and ligated with OriE and cosλ as well as *hyg^r^* and *sacB*, obtained from pYUB1474 (49), to generate AES (allelic exchange substrate). SnaBI-linearized AES was used to generate *RvΔembR* mutant as described previously (50). Hygromycin-resistant colonies were screened to replace the *embR* gene at its native loci with the help of multiple PCRs and Western blots.

### Generation of plasmid DNA constructs and strains.

pNiT-1 and pNiT-ET plasmids were a kind gift from Christopher M. Sassetti and Eric Rubin, respectively (51). pQEII-embR, used for protein expression and purification, was generated by amplifying *embR* from *H37Rv* genomic DNA and cloned into pQEII vector using NdeI/HindIII sites. *embR* was further subcloned into pFICTO to generate pF-F-embR (with an N-terminal 3×FLAG tag). The constructs were further electroporated into *RvΔembR* mutant to generate *RvΔembR*::F*embR* mutant. To generate *embR* complementation constructs with its native promoter, *embR* without stop codon and 500 bp immediately upstream of the gene was PCR amplified and cloned into ScaI-HindIII sites of pFICTO vector to generate pF-embR-HA (with a C-terminal 3× hemagglutinin [HA] tag). pF-embR-HA construct was electroporated into *RvΔembR* mutant to generate *RvΔembR*::*embR* mutant. For the reporter assays, the 500-bp region upstream of *hspX* was cloned in ScaI-NdeI sites of pSW1-Luciferase construct (unpublished data) using ScaI-NdeI sites to generate pSW1-hsp-luc. The construct was electroporated into *Rv*, *RvΔembR*, and *RvΔembR*::*embR* strains.

### Growth rate kinetics.

Exponential-phase cultures of *Rv*, *RvΔembR*, and *RvΔembR*::*embR* strains grown in Middlebrook 7H9-ADC medium were seeded at an optical density at 600 nm (OD_600_) of ~0.05 in 7H9/Sauton’s medium. OD_600_ was monitored every 24 h, and the CFU were enumerated by serially diluting the cultures and plating them on 7H11 agar.

### Scanning electron microscopy and transmission electron microscopy.

Mycobacterial strains cultured in 7H9-ADC medium and 10-mL cultures at an OD_600_ of ~0.6 were harvested. SEM and TEM were performed as previously described (50). Briefly, for TEM, cells were fixed, gradually dehydrated, and polymerized using Epson 812 resin; ~63-nm sections were cut using an ultramicrotome (Leica) and subsequently stained using uranyl acetate and lead citrate for visualization under a Tecnai G2 20 twin (FEI) transmission electron microscope (52). Cell length, width, and thickness were quantified with the help of Smart TIFF software and Carl Zeiss Tiff Annotation Editor.

### *In vitro* stress susceptibility assays.

Susceptibility of *Rv*, *RvΔembR*, and *RvΔembR*::*embR* strains to various *in vitro* stresses was determined as previously described (53). Briefly, log-phase bacillary cultures were washed with PBST_80_ (phosphate-buffered saline and 0.05% Tween 80) and seeded at an OD_600_ of ~0.05 in 7H9-ADS (ADC without catalase) medium containing 50 μM cumene hydroperoxide for a day. CFU were enumerated by plating on 7H11-OADS (OADC without catalase) agar plates. For assessing the susceptibility to surfactant stress, bacterial cells were cultured in 7H9-ADC medium containing 0.05% SDS for 3 h. The survival of strains was also examined upon the addition of 1 mM DTT for 24 h. To induce nitrosative stress, cultures were grown in 7H9-ADC medium (pH 5.5, acidified with the help of HCl) in the presence and absence of 3 mM NaNO_2_ for 48 h. Harvested cells were washed with PBST_80_, and serially diluted cultures were spotted on 7H11-OADC agar to enumerate CFU.

### Hypoxia experiment.

*In vitro* hypoxia stress was assessed through modified Wayne’s hypoxia model as described earlier (54). Briefly, bacterial strains were inoculated in 7H9-ADC at an OD_600_ of ~0.1; 1.5 μg/mL of methylene blue was added to a visual redox indicator. The experiment was carried out in tightly sealed glass tubes with 15% headspace at 37°C without agitation. Bacterial cells were serially diluted and plated on 7H11-OADC agar to enumerate the CFU.

### Peritoneal macrophage infections.

Peritoneal macrophages were isolated from BALB/c mice and cultured as previously described (55). Briefly, 4- to 6-week-old mice were injected with 4% thioglycolate solution (Difco) in the peritoneal cavity. After 96 h, the mice were euthanized, and isolated peritoneal macrophages were seeded at a density of 1 million cells/well in a 12-well tissue culture plate in RPMI 1640 with 10% fetal bovine serum. Single-cell suspensions of the mycobacterial *Rv*, *RvΔembR*, and *RvΔembR*::*embR* strains were prepared to infect the peritoneal macrophages at a multiplicity of infection (MOI) of 1:10 (cell-bacteria). Cells were lysed with SDS (0.05%), and CFU were enumerated.

### Murine infection experiment.

Exponential-phase cultures of *Rv* and *RvΔembR* mutant were washed and resuspended in the neutral saline, and single-cell suspensions were prepared. BALB/c mice (4 to 6 weeks old) were housed in the Tuberculosis Aerosol Challenge Facility (TACF) at International Centre for Genetic Engineering and Biotechnology (ICGEB), New Delhi. Mice in groups (*n* = 6 to 7) for each time point were challenged with the bacterial strains by aerosol to implant 100 bacilli per mouse. Bacillary deposition in the lungs was assessed 24 h postinfection. Four and 8 weeks postinfection, the bacterial survival was examined in both lungs and spleen. The fixed tissues were also examined for histopathology and granuloma scoring by hematoxylin and eosin staining, as described previously (54).

### RNA isolation and qRT-PCR analysis.

Isolation of RNA was performed using TRIzol reagent (Ambion). Cell numbers equivalent to an OD of 10 for *Rv*, *RvΔembR*, and *RvΔembR*::*embR* strains were harvested and resuspended in TRIzol reagent. The cells were lysed using 0.1-mm zirconium beads, following which RNA was extracted through chloroform, precipitated, and washed using 70% ethanol (prepared in diethyl pyrocarbonate-water). The RNA was resuspended in the nuclease-free water and further purified using the RNeasy minikit (Qiagen). The samples were treated with DNase I to remove any leftover DNA traces. cDNA was synthesized using the iScript cDNA synthesis kit (Bio-Rad) according to the manufacturer’s protocol. Gene expression with gene-specific primers (Table 2) was measured with iTaq Universal SYBR green supermix (Bio-Rad). The gene induction ratio was normalized to 16S rRNA, and results were analyzed using the ΔΔ*C_T_* method.

### RNA-seq analysis.

RNA isolated from two biological replicates of either normoxic or hypoxic samples of *Rv* and *RvΔembR* strains were used to prepare the library and sequenced on the Illumina platform. The reads obtained were assessed for quality control using the FastQC package, followed by alignment with the M. tuberculosis genome and annotation downloaded from Ensembl (56) using bowtie2 (57). Htseq-count was used to count the number of reads mapped per gene (58). DEseq2 was used to get normalized counts and for analysis of differential expression (59). Figures were made using custom scripts in python3.

Gene ontology was performed for genes with *P*adj of <0.1 and 0.4 log_2_ fold change using PANTHER version 14 (60). The obtained results were graphically represented using R.

### Luciferase assays.

*Rv*, *RvΔembR*, and *RvΔembR*::*embR* strains electroporated with pSW1-hspX-luc construct and pSW1-devS-luc construct were grown in 7H9-ADC medium until mid-log phase. Cells were harvested at an OD_600_ of ~0.6, and the lysates were prepared in the lysis buffer (25 mM Tris-phosphate, pH 7.8, 10% glycerol, 2 mM DTT, 1% Triton X-100, protease inhibitor). Luciferase assay using Luciferase assay kit (Promega E1500) was performed per the manufacturer’s protocol. Briefly, a defined volume of luciferase assay reagent (LAR) was added to1 μg of whole-cell lysates from each strain, and luminescence was measured using a Berthold luminometer.

### MIC.

Resazurin microtiter assay (61) was used to determine the MIC in M. tuberculosis strains. Briefly, antibiotics were serially diluted in 7H9-ADC medium (without Tween 80) with appropriate controls in a flat-bottom 96-well plate. Mid-log-phase bacilli from *Rv*, *RvΔembR*, and *RvΔembR*::*embR* strains were diluted to an OD_600_ of ~0.006 in 7H9-ADC, and 100 μL from each strain was added to the plate. A volume of 20 μL of resazurin solution (0.02%) was added after 5 days of incubation at 37°C. Bacterial growth was indicated by a color transition from blue to pink the following day. The antibiotic concentration that prevented this color change was defined as the MIC.

### Lipidomics analysis.

*Rv*, *RvΔembR*, and *RvΔembR*::*embR* strains were grown in 7H9-ADC either under regular growth conditions or subjected to hypoxic stress. Cells equivalent to an OD_600_ of ~6 were washed thrice with cold, sterile PBS and resuspended in 1 mL PBS. The resuspended cells were transferred into a glass vial. The cellular lipids were extracted using an established protocol (33) by adding 3 mL of 2:1 (vol/vol) chloroform-methanol mixture. Samples were vortexed and centrifuged at 2,800 × *g* (15 min at 4°C), and the organic layer was collected. The remaining aqueous layer was acidified (2% formic acid) and reextracted using chloroform (2 mL) to enrich phospholipids. The samples were once again centrifuged, and the organic layer was collected. The organic layers were pooled and dried under a stream of nitrogen gas at room temperature. The dried pellets were resuspended 200 μL of 2:1 (vol/vol) chloroform-methanol and subjected to semiquantitative (relative quantification) information-dependent acquisition (IDA)-mediated LC-MS/MS analysis (34, 35) on a Sciex X500R quadrupole time-of-flight (QTOF) mass spectrometer fitted with an Exion ultrahigh-performance LC system. The LC was performed on a Gemini 5U C_18_ column (5 μm, 50 by 4.6 mm; Phenomenex) coupled to a Gemini guard column (4 by 3 mm; Phenomenex security cartridge; Phenomenex).

### Phosphorylation study and mass spectrometry analysis.

We used the previously described pETDuet-1 system for coexpressing EmbR and M. tuberculosis STPKs (62). pDUET-STPK constructs, where STPKs were cloned with N-terminal MBP tag into MCS2, were described earlier (62). *embR* amplified using *Rv* genomic DNA was cloned into duet-STPK constructs using the HindIII site in MCS-I site such that the protein was expressed with N-terminal hexa-His tag. Constructs were further transformed in E. coli B21 DE3 Codon Plus, and the expression of His-EmbR and MBP-STPKs was confirmed by Western blotting. His-tagged EmbR was pulled down using Ni^2+^-nitrilotriacetic acid agarose beads and processed for mass spectrometry using the in-gel digestion method (63).

For identifying EmbR phosphorylation sites in M. tuberculosis, pNit-pknB, pNit-pknD, pNit-pknH, and pNit-pknF constructs were electroporated into *RvΔembR*::*F-embR* strain. FLAG-EmbR was immunoprecipitation (IP), and 1/10 of the immunoprecipitated samples was probed with α-FLAG antibodies and 9/10th of the sample was resolved on SDS-PAGE and probed with α-p-Thr antibodies. To identify EmbR phosphorylation sites under specific conditions, whole-cell lysates (WCLs) were prepared from *Rv* grown under various stress conditions. EmbR protein in the lysates was immunoprecipitated using mouse α-EmbR polyclonal antibodies; 1/10th of the IP sample was probed with α-EmbR antibodies, and 9/10th of the sample was probed with α-p-Thr antibodies.

Sample resolved on the gel was processed for mass spectrometry analysis as described earlier (64). Briefly, sample were treated with Tris(2-carboxyethyl)phosphine-HCl (TCEP) and iodoacetamide (IAA), followed by trypsin digestion (1:100, wt/wt, trypsin-protein). Tryptic peptides were desalted using C_18_ columns, dried, and resuspended in the reconstitution buffer (5% organic solution consisting of 80% acetonitrile in 0.1% formic acid and 95% water). Liquid chromatography-mass spectrometry was performed using LTQ Orbitrap Velos, and the analysis was performed with the help of Proteome Discoverer Software Suite (version 1.3). Samples were searched with SEQUEST by keeping methionine oxidation and carbamidomethylation of cysteine (C) as constant modification and serine (S), threonine (T), and tyrosine (Y) phosphorylation as dynamic modifications. All the spectra were searched against the M. tuberculosis UniProt database.

### Statistical analysis.

The results’ significance was analyzed using the Student’s *t* test (two-tailed, unpaired, nonparametric) unless otherwise specified. The results were plotted on GraphPad Prism version 9.0 and modified with Adobe Photoshop and Adobe Illustrator.

### Ethics.

Animal experiment protocols were reviewed and approved by the Institutional Animal Ethics Committee of the National Institute of Immunology, New Delhi, India (IAEC approval number 462/18). The experiments were carried out per the guidelines issued by the Committee for the Purpose of Control and Supervision of Experiments on Animals (CPCSEA), Government of India.

### Data availability.

The source data for all the figures can be obtained from the corresponding author upon request. RNA sequencing data were deposited in the GEO repository with GEO accession number GSE154673.
