# Supplementary figures and images for: Mycobacterium tuberculosis Transcription Factor EmbR Regulates the Expression of Key Virulence Factors That Aid in Ex Vivo and In Vivo Survival
Source: mBio. 2022 Apr 26;13(3):e03836-21. doi: 10.1128/mbio.03836-21 (PMC9239209; doi:10.1128/mbio.03836-21)

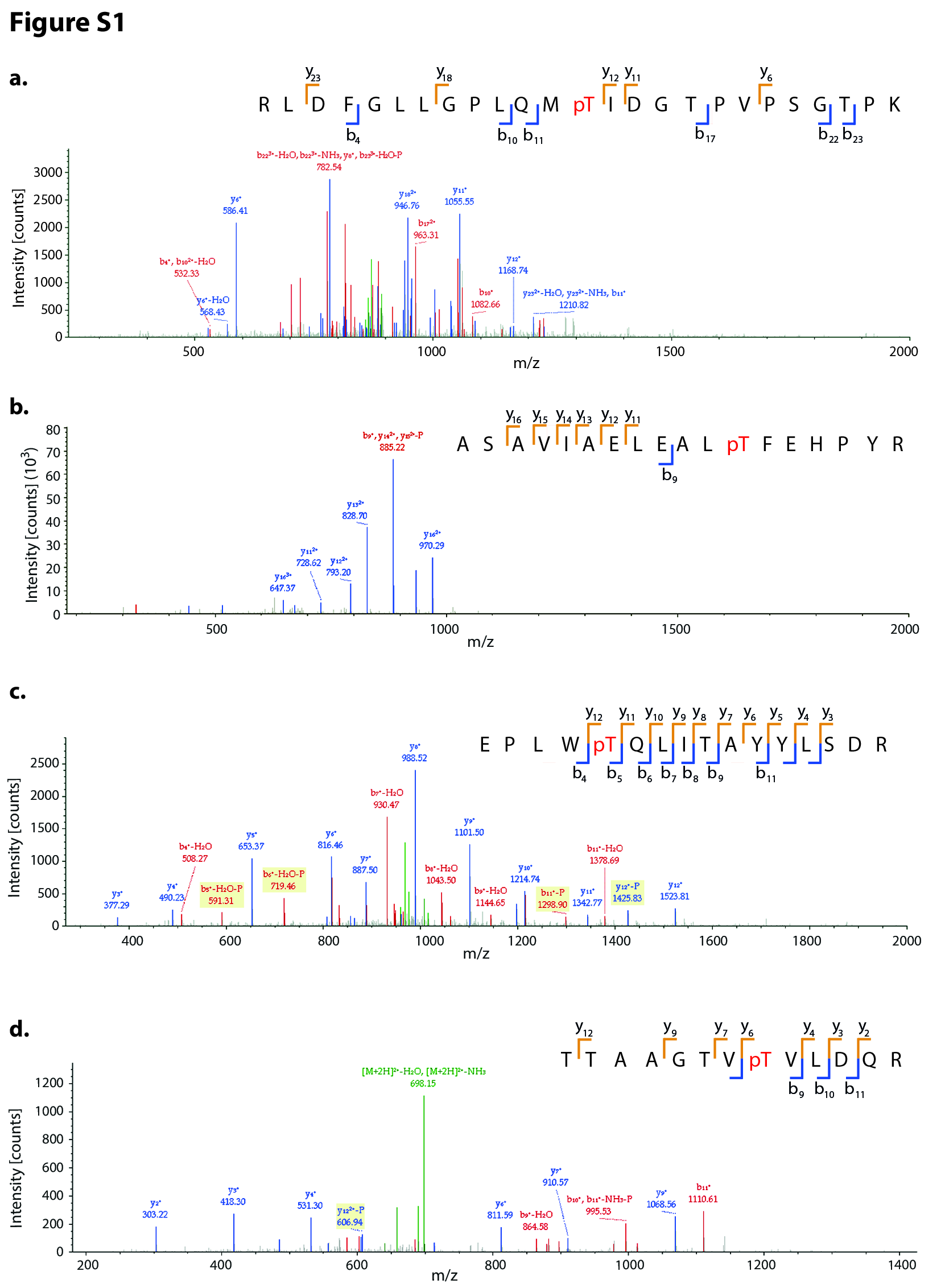

Supplement: FIG S1 [file mbio.03836-21-s0009.tif]

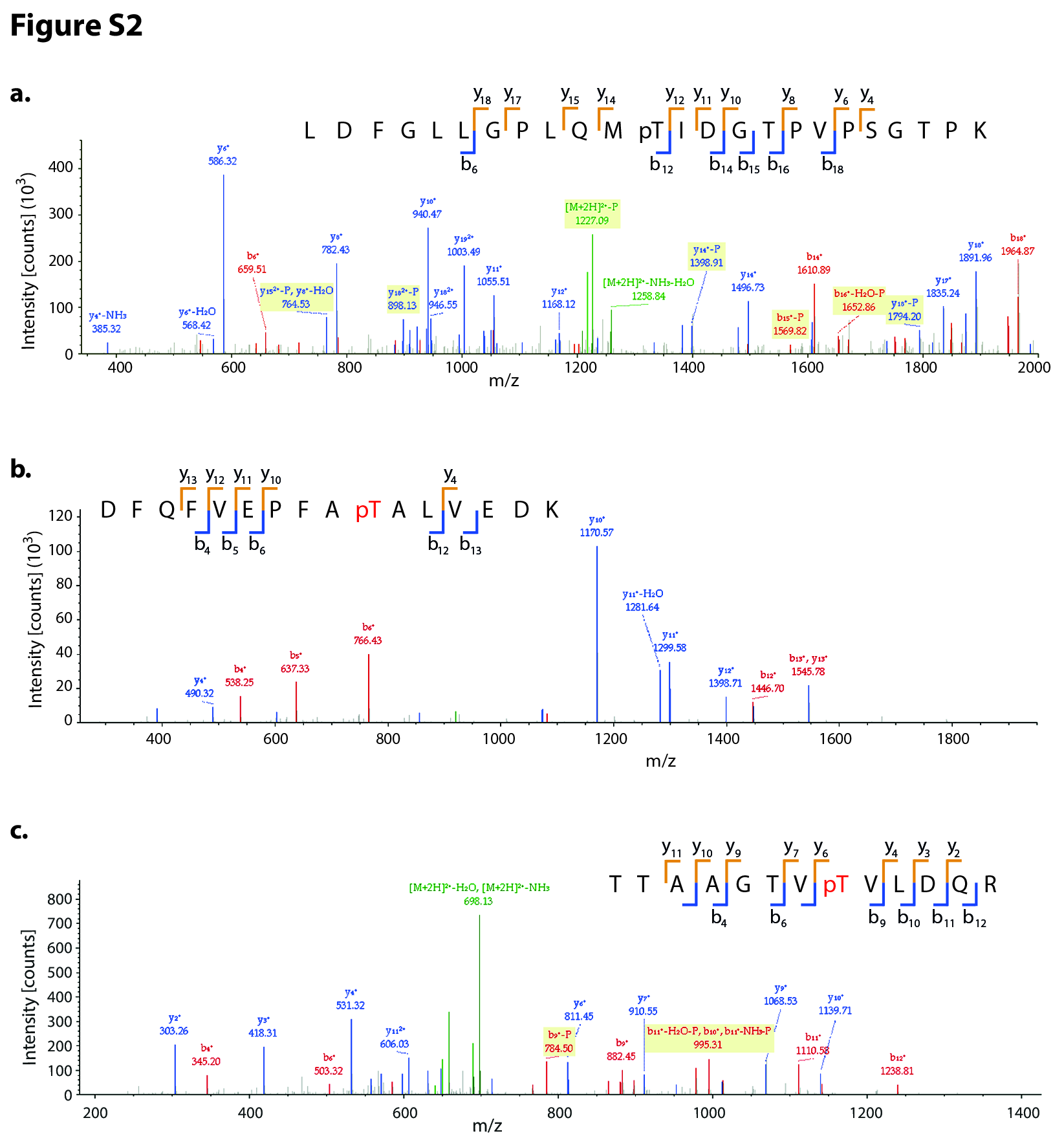

Supplement: FIG S2 [file mbio.03836-21-s0001.tif]

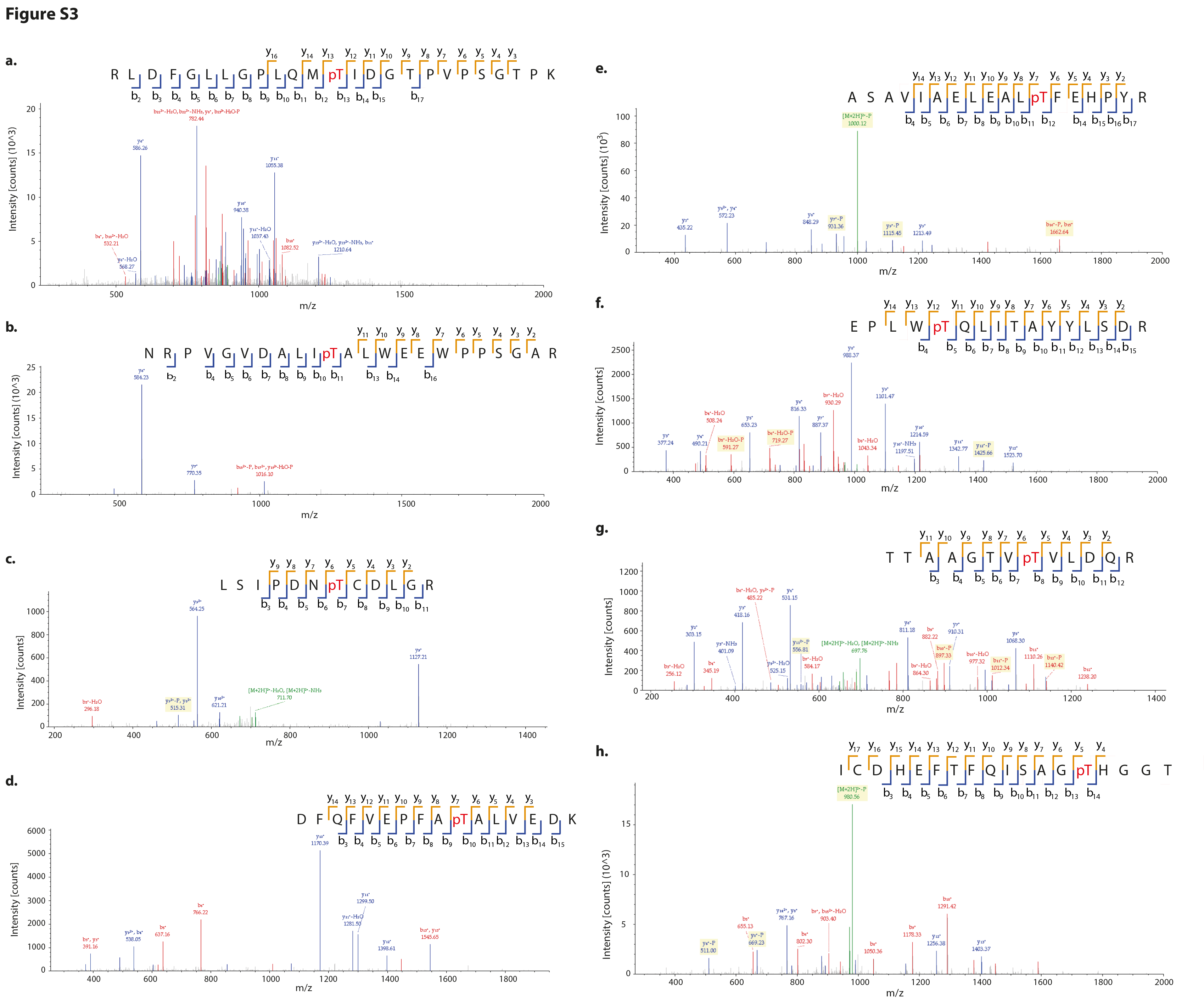

Supplement: FIG S3 [file mbio.03836-21-s0002.tif]
